# NF-Y Binding Site Architecture Defines a C-Fos Targeted Promoter Class

**DOI:** 10.1371/journal.pone.0160803

**Published:** 2016-08-12

**Authors:** Martin Haubrock, Fabian Hartmann, Edgar Wingender

**Affiliations:** Institute of Bioinformatics, University Medical Center Göttingen (UMG), Georg-August-University Göttingen, Goldschmidtstrasse 1, 37077 Göttingen, Germany; Università degli Studi di Milano, ITALY

## Abstract

ChIP-seq experiments detect the chromatin occupancy of known transcription factors in a genome-wide fashion. The comparisons of several species-specific ChIP-seq libraries done for different transcription factors have revealed a complex combinatorial and context-specific co-localization behavior for the identified binding regions. In this study we have investigated human derived ChIP-seq data to identify common cis-regulatory principles for the human transcription factor c-Fos. We found that in four different cell lines, c-Fos targeted proximal and distal genomic intervals show prevalences for either AP-1 motifs or CCAAT boxes as known binding motifs for the transcription factor NF-Y, and thereby act in a mutually exclusive manner. For proximal regions of co-localized c-Fos and NF-YB binding, we gathered evidence that a characteristic configuration of repeating CCAAT motifs may be responsible for attracting c-Fos, probably provided by a nearby AP-1 bound enhancer. Our results suggest a novel regulatory function of NF-Y in gene-proximal regions. Specific CCAAT dimer repeats bound by the transcription factor NF-Y define this novel cis-regulatory module. Based on this behavior we propose a new enhancer promoter interaction model based on AP-1 motif defined enhancers which interact with CCAAT-box characterized promoter regions.

## Introduction

Transcription factors (TFs) are proteins that control gene expression through a variety of mechanisms such as enhancing the efficiency of the basal transcription complex to assemble or re-model chromatin. Most of them act by recognizing cis-regulatory elements or TF binding sites (TFBS) in gene proximal (promoter) or distal (enhancer) regions in a sequence-specific way. Each regulatory region is defined by an array of such TFBSs. The cooperative binding of multiple TFs to these closely located TFBSs determines the transcription activity of their target genes [1, 2]. Potential TFBSs can be found everywhere in the genome, but only a minority of them appears to be functional in a given cellular context. Moreover, the activity of a proximal or a distal region in a certain cellular context is associated with its epigenetic status such as DNA- or histone modification, and can be monitored by its sensitivity against DNase I attack [3, 4]. However, it is unclear whether the binding of a TF is the prerequisite or the consequence of an epigenetic modification. Different genomic studies have shown the importance of so-called pioneer- or master-TFs in this process [5, 6]. In any case, the switching between the inactive and the active state of a regulatory region must be triggered by an event that involves a signal encoded in its sequence [7].

The ChIP-seq technology is widely used to systematically identify gene regulatory regions interacting with a known TF. ChIP-seq experiments combine chromatin immunoprecipitation with high-throughput sequencing to reveal TF-bound regions in a genome-wide fashion [8]. The Encyclopedia of DNA Elements (ENCODE) project provides a large collection of human related ChIP-seq data for more than 100 different TFs as well as their binding behavior in different cell types/cell lines [9, 10]. Based on this rich information resource some important observations have been made regarding the cooperation of TFs: (a) Transcription factors co-localize (overlap of ChIP-seq regions for different TF) in a complex combinatorial and context-specific fashion; different groups of TFs tend to co-localize at distinct sets of proximal and distal regulatory regions [10]. (b) Extensive changes of these TF co-localizations within a cell exposed to different conditions or across multiple cell types demonstrate the complexity of the gene regulatory syntax [11]. (c) ChIP-seq experiments cannot distinguish between direct and indirect TF occupancy [4, 12]. (d) The occurrence of general “non-targeted TF motifs”, i.e. motifs not known to be associated with the precipitated TF, seems to be a “systematic component of ChIP-seq data sets” [13].

The missing capability of ChIP-seq experiments to distinguish between direct and indirect TF binding is a notable fact which explains why the interpretation of ChIP-seq-derived regulatory regions is still a challenging task. For example, the nuclear glucocorticoid receptor (GR) and the activator protein 1 (AP-1) have been shown to interact with each other in two different modes. GR can interact with AP-1 in a DNA-dependent mode using two adjacent composite regulatory elements for the TFs GR and AP-1. Alternatively, the GR can interact with AP-1 at non-composite elements through protein-protein interaction (tethering). These regions contain an AP-1 binding motif (TFBS), but no corresponding GR binding motif [14].

Complex and dynamic co-localization patterns were described for a set of TFs related to ChIP-seq experiments done in one cell type under changing conditions or across different cell types. As an example, it was shown that proximal and distal c-Fos precipitated ChIP-seq regions are characterized by different sets of co-localized TFs [10]. For the cell line K562 it was shown that this bisection of c-Fos ChIP-seq regions is an oversimplification [11]. Here, at least least five different sub-classes of co-associations with the transcription factor c-Fos were described. The c-Fos protein is part of a heterodimer called AP-1. For DNA-binding, c-Fos (or one of its paralogs FosB, Fra-1 or Fra-2) has to heterodimerize with a member of the structurally and functionally related Jun protein family (Jun, JunB and JunD) [15, 16]. AP-1 regulates a number of cellular processes including proliferation, differentiation, apoptosis and transformation [17]. Therefore, an AP-1 derived regulatory influence should be accompanied by a defined co-localization profile for Fos and Jun protein family members [10, 11]. The majority of all c-Fos precipitated ChIP-seq regions in the K562 cell line showed a c-Fos and JUND co-localization profile [11]. Interestingly, a subset of co-localized c-Fos and NF-YA/NF-YB ChIP-seq regions in K562 cell lines exists that does not show any significant co-localization pattern to the Jun protein family. Another study reported that NF-YB precipitated regions extensively co-associate with c-Fos typically at those regulatory regions that lack an AP-1 motif [18]. More recently it was shown that the CCAAT box motif is the primary binding site in these co-localizing c-Fos and NF-YB ChIP-seq regions [19]. In addition the authors observed a distance relationship between two CCAAT box motifs in the overlap of c-Fos and NF-YB bound regions [19].

In the light of these findings, we made an attempt to revisit the existing c-Fos and NF-YB ChIP-seq data done in the ENCODE framework with regard to predominant sequence motifs. In this study, we present a systematic analysis of ChIP-seq data for the transcription factor c-Fos in the cell lines HUVEC, HeLa S3, K562, and GM12878. We developed a workflow based on Receiver Operator Characteristic (ROC) curves to identify important TFBS motifs for each proximal and distal ChIP-seq data sets separately. Using cell-type specific non-overlapping DNase-seq data sets as a negative control, we were able to identify the main regulators for each binding regions. We found that in four different cell lines, c-Fos precipitated proximal (promoter) and distal (enhancer related) genomic intervals show clear prevalences for either AP-1 motifs or CCAAT boxes as known binding motifs for the transcription factor NF-Y, which occur in a mutually exclusive manner. Based on this behavior we propose a new enhancer promoter interaction model based on AP-1 motif defined enhancers which interact with CCAAT-box characterized promoter regions. We show that this type of interaction is specific for genes associated with a certain group of biological processes of Gene Ontology.

## Results

### Many c-Fos-ChIP-seq data sets reveal CCAAT-motif rather than an AP-1 consensus as dominant motif

**Fig 1:**
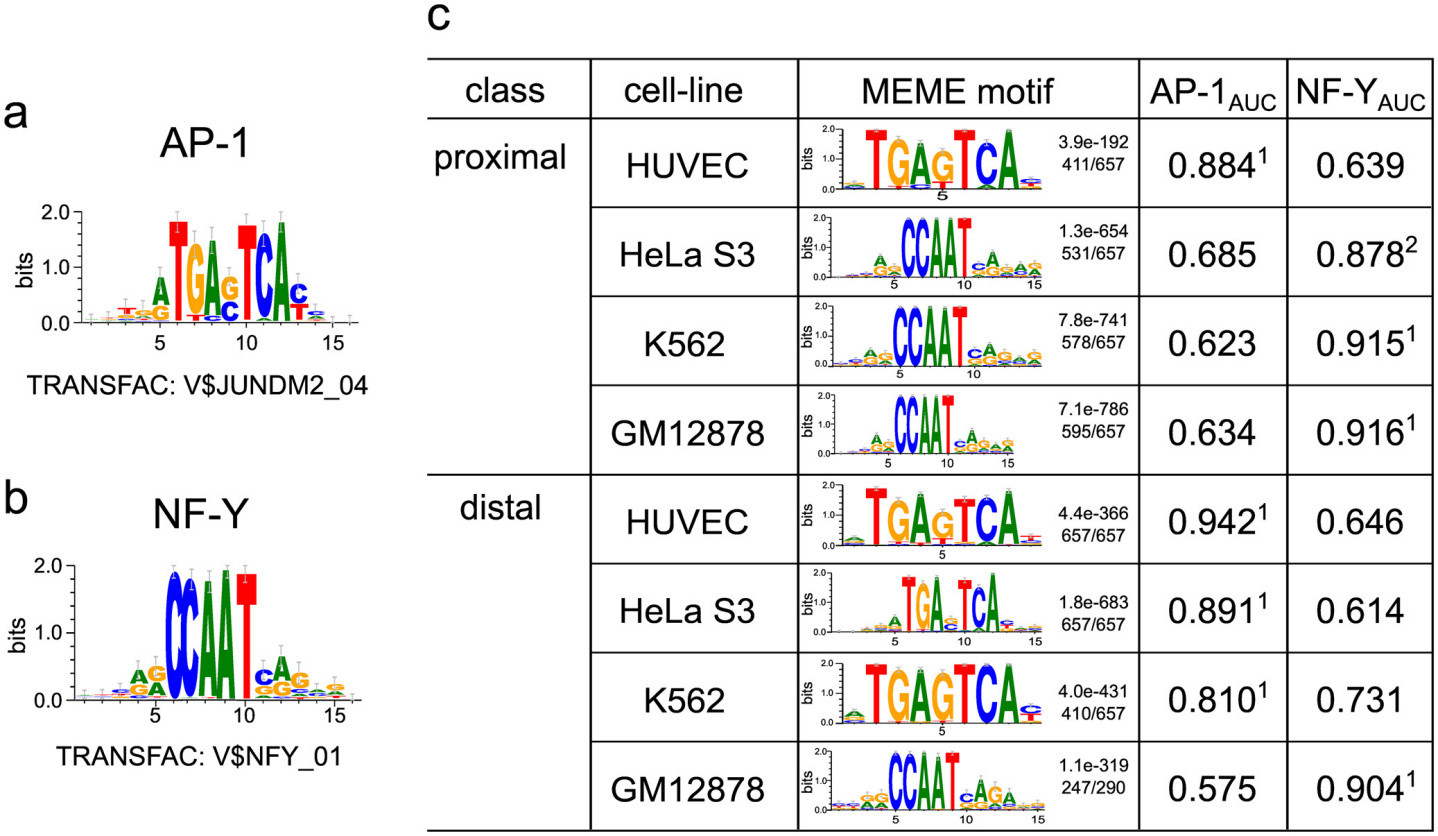
Prevalent motifs in ChIP-seq intervals precipitated with c-Fos antibodies. (**a**): Logo plot representing the AP-1 PWM from the TRANSFAC database. (**b**) Logo plot for the NF-Y PWM from TRANSFAC. (**c**) Summary of the top-ranking motifs obtained with MEME for proximal and distal sequence sets from the four cell lines under study. The rank of the two PWMs shown in (**a**) and (**b**) sorted by the AUC values for all 2176 TRANSFAC-related PWMs are given as superscript indices in this Table (see Methods for details).

We launched a systematic analysis of c-Fos ChIP-seq data that are available in the ENCODE repository [10], using the PWM library of the TRANSFAC database [20]. When we started this study, corresponding data were available for four different cell lines: HUVEC, HeLa S3, K562, and GM12878. Since it is also known that enhancer and promoter regions may behave differently, we analyzed separately the ChIP-seq intervals that are located distally or proximally to annotated transcription start sites (TSS) in each cell line (see Methods for details). The analysis was done (a) by recording Receiver Operating Characteristic (ROC) curves with TRANSFAC matrices in order to identify the best classifier compared to non-overlapping cell-type specific DHS regions for each sequence set or (b) by applying the MEME program to identify *de novo* the most prevalent sequence motif in each ChIP-seq data set (see Methods for details). The Fig 1 summarizes our findings. We found that besides the AP-1 motif, which was expectedly indicative for some of the data sets, a couple of cell line specific ChIP-seq regions were characterized by position weight matrices (PWMs) for the transcription factor NF-Y, which binds to CCAAT boxes [21]. For the ROC curves obtained with two representative matrices for these two TFs, we measured the Area Under the Curve (AUC) values (see Methods and S1 and S2 Figs for details). The MEME analysis confirmed our ROC/AUC results. The top enriched motif for each sequence set is shown in Fig 1 (see Methods for details and S3–S6 Figs).

The ROC curves and the corresponding MEME analysis proved that (a) proximal and distal regions differ in their prevalence of either AP-1 or NF-Y binding motifs, and (b) this feature is also different among the four cell lines. In human umbilical vein endothelial cells (HUVEC), both proximal and distal c-Fos-bound regions are enriched for potential AP-1 sites. Both types of ChIP-seq intervals (distal and proximal) from GM12878 (a human lymphoblastoid cell line) harbor CCAAT boxes as the most characteristic trait (see also S2 and S6 Figs). The low performance of this motif in discriminating either data set against the background sequences is demonstrated by the small AUC values (distal: 0.634, proximal: 0.575, see Fig 1 and S1 and S2 Figs for details). It should be noticed, however, that the total number of c-Fos bound distal regions in GM12878 is very small (Table 1, S1 Table). More complex are the situations in the case of HeLa S3 and K562 cells. While the distal intervals revealed recognizable AP-1 motifs, the proximal regions showed a clear preponderance of potential NF-Y binding sites, i.e. CCAAT boxes (S1 and S2 Figs). The less pronounced motif, AP-1 in proximal and CCAAT boxes in distal regions, are close to the background noise and do not even show up among the top 5 motifs detected by MEME (S4 and S5 Figs).

**Table 1:** Overlap of c-Fos and NF-YB precipitated ChIP-seq intervals. Genomic proximal (p) and distal (d) intervals precipitated with anti-c-fos and and-NF-YB antibodies and documented in the ENCODE data repository were analyzed for overlaps. The number of unique genomic intervals and their percentage in the union set of both groups are shown (see Methods for details).

Cell line	type	c-Fos	c-Fos+NF-YB	NF-YB
HeLa S3	p	166 (7.6%)	491 (22.3%)	1540 (70.1%)
	d	4104 (63,3%)	351 (5.4%)	2030 (31.3%)
K562	p	401 (14.9%)	1511 (56.2%)	779 (28.9%)
	d	1745 (28.4%)	904 (14.7%)	3488 (56.8%)
GM12878	p	184 (6.0%))	1101 (35.8%)	1790 (58.2%)
	d	39 (1.0%)	251 (5.2%)	4560 (94.0%)

### Co-localization analysis of c-Fos- and NF-YB-targeted ChIP-seq regions

Since we have observed that a large number of c-Fos-targeted genomic intervals, in particular proximal intervals, exhibit NF-Y binding motifs rather than the expected AP-1 motifs, we checked whether they have already been evidenced as bound by the transcription factor NF-Y. For three out of four cell lines investigated here, ChIP-seq data sets for the subunit NF-YB were available in the ENCODE repository (HeLa S3, K562 and GM12878). It was shown that NF-YB precipitated ChIP-seq regions define NF-Y binding sites [18]. We therefore checked them for overlaps with c-Fos-bound intervals (see Methods for details; S1 Table). The Table 1 summarizes the cell-type specific co-localization patterns of these two TFs separately for proximal (p) and distal (d) genomic intervals.

**Fig 2:**
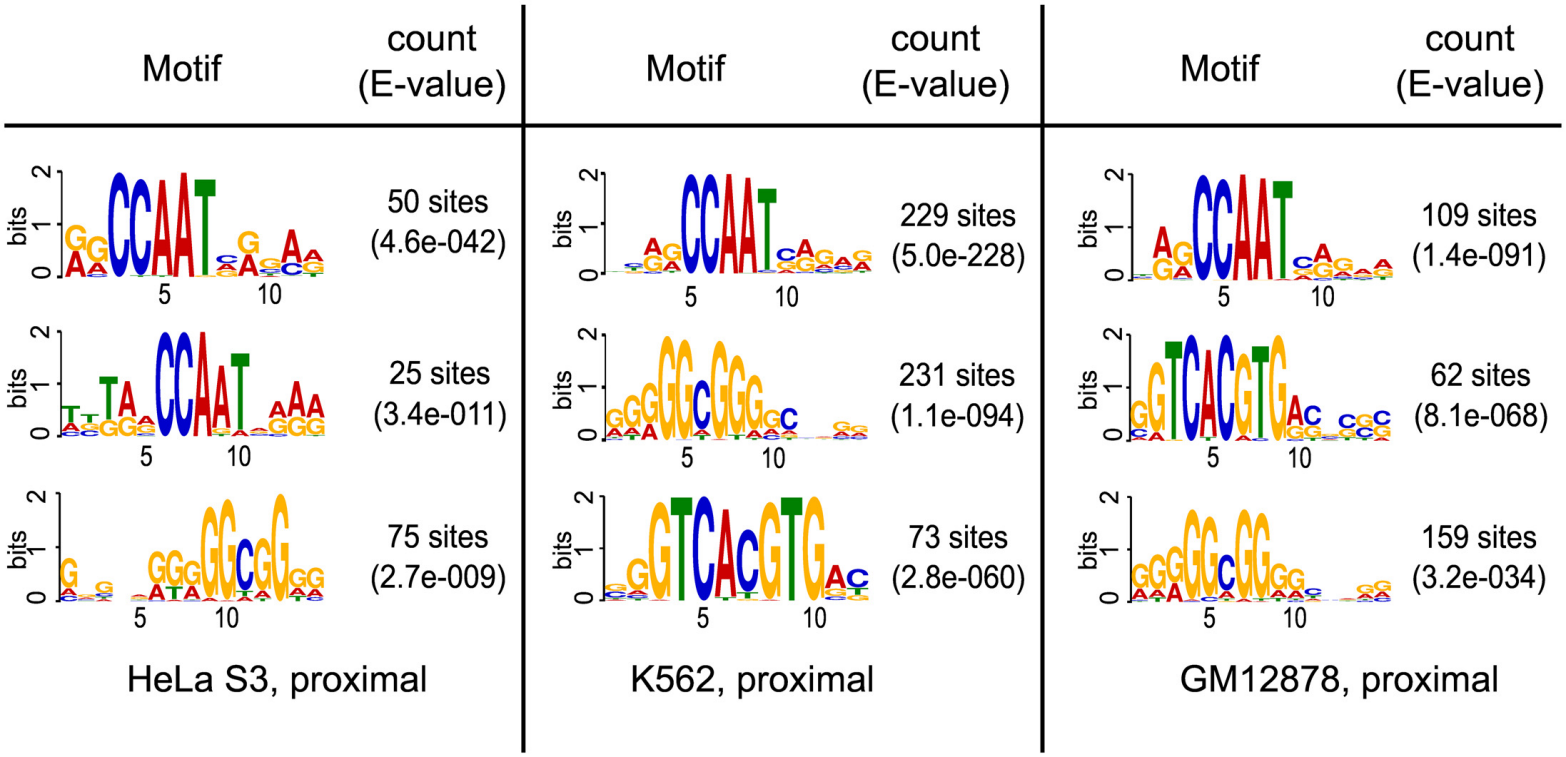
Enriched motifs in the c-Fos(only) targeted proximal sequences lacking an AP-1 motif. Two instances of a NF-Y related CCAAT motif are observed in the three different cell lines. Additionally we observe an Sp1 related motif (GGCGG). Finally, an E-box motif was found in the proximal regions for the K562 and GM12878 cell line.

We found that most of the proximal c-Fos-targeted regions were also bound by NF-YB (HeLa S3: 74.7%; K562: 79.0%; GM12878: 85.7%). All of them exhibited a CCAAT box as prevalent motif (data not shown). Among those intervals that interact with c-Fos(only), i.e. not with NF-YB, only a subset harbors a high-scoring AP-1 motif, that could be identified by TRANSFAC matrix V$JUNDM2_04. For sake of conciseness, we denote in the following genomic intervals that are targeted by c-Fos(only) and do have an AP-1 motif above the optimized threshold with c-Fos(only, proximal, AP-1+). The regions that lack such a motif are labeled with c-Fos(only, proximal, AP-1−). The portion of c-Fos(only, proximal, AP-1+) intervals varies largely between the three cell lines (51% for HeLa S3, to 19% for K562 down to 3% in GM12878 cells). Motif discovery using MEME confirmed the predominant role of AP-1 consensus motifs in these c-Fos(only, proximal, AP-1+) sequences (S7 Fig). In addition, when applying MEME to the cell-type specific c-Fos(only, proximal, AP-1−), we found the CCAAT box motif as the dominant motif in these proximal data sets (Fig 2). Tables 2 and 3 are summarizing the proportions of AP-1 and CCAAT box motifs in more detail. The two tables demonstrate that the main trait of c-Fos(only, proximal) genomic intervals is the presence of CCAAT box motifs while the c-Fos(only, distal) genomic intervals are mainly characterized by an AP-1 motifs. This disproportion is demonstrated by the corresponding Odds and Odds ratios for all three cell lines.

**Table 2:** c-Fos bound proximal regions are depleted of AP-1 motifs. Proportion of AP-1 motif occurrence in proximal and distal genomic ChIP-seq intervals bound by c-Fos, but not by NF-YB are shown. Proximal (p) and distal (d) genomic intervals targeted by c-Fos, but not by NF-YB (column c-Fos of Table 1) differ significantly in the occurrence of AP-1 motifs detected by an AP-1 PWM (Fig 1); AP-1+: number of intervals with, AP-1−: without a detectable AP-1 motif. In the last column the Fisher’s Exact Odds Ratios (OR, bold) and the corresponding P-values (in parentheses) are given.

Cell line	type	AP-1+	AP-1−	Odds	Odds Ratio (P-value)
HeLa S3	**p**	85	81	1.0494	**0.276**
	**d**	3478	626	5.556	(1.366e-14)
K562	**p**	77	324	0.238	**0.038**
	**d**	1505	240	6.271	(5.428e-150)
GM12878	**p**	5	179	0.028	**0.008**
	**d**	31	8	3.875	(2.200e-25)

**Table 3:** c-Fos bound proximal regions are enriched by CCAAT box motifs. Proportion of CCAAT box motif occurrence in proximal and distal genomic ChIP-seq intervals bound by c-Fos, but not by NF-YB are shown. Proximal (p) and distal (d) genomic intervals targeted by c-Fos, but not by NF-YB (column c-Fos of Table 1) differ significantly in the occurrence of the CCAAT box motifs detected by an NF-Y PWM (Fig 1); CCAAT+: number of intervals with, CCAAT−: without a detectable CCAAT box motif. In the last column the Fisher’s Exact Odds Ratios (OR, bold) and the corresponding P-values (in parentheses) are given.

Cell line	type	CCAAT+	CCAAT−	Odds	OR (P-value)
HeLa S3	**p**	52	114	0.325	**49.890**
	**d**	37	4067	0.009	(4.373529e-53)
K562	**p**	203	198	1.0253	**32.622**
	**d**	53	1692	0.031	(1.179e-118)
GM12878	**p**	98	86	1.139	**42.838**
	**d**	1	38	0.026	(1.022e-79)

**Fig 3:**
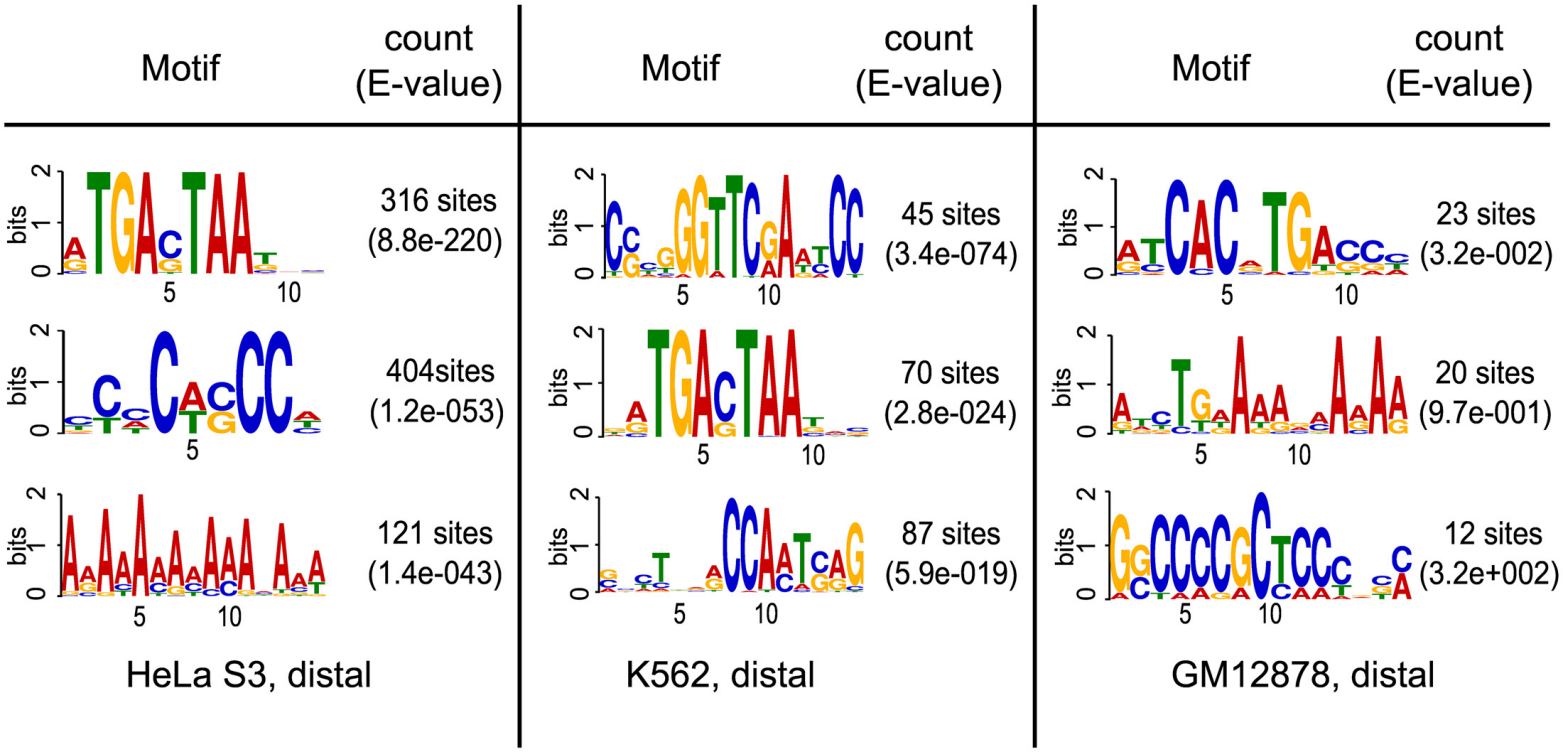
Enriched motifs in the c-Fos(only) targeted distal sequences lacking an AP-1 motif. In contrast to the proximal genomic intervals no enrichment of CCAAT box motifs was found in the distal regions. Instead, an alternative AP-1 motif was found enriched in HeLa S3 and K562 distal intervals.

When we analyzed the cell-type specific co-localization pattern of c-Fos and NF-YB targeted regions for distal intervals, the picture clearly differed from that of the proximal regions. Only 8% of c-Fos targeted distal regions overlapped with NF-YB regions in HeLa S3. This portion was larger for K562 (34.1%), and the largest co-localization pattern was observed for the GM12878 cell line (86.6%). When we assessed the amount of high-scoring AP-1 motifs in distal regions we found much higher proportions of c-Fos(only, distal, AP-1+) than in the proximal regions (84.5% for HeLa S3, 86.2% for K562, and 79.5% for GM12878). Motif discovery using MEME verified the dominant role of AP-1 motifs in this group (data not shown). In contrast to the proximal intervals we found no enrichment of CCAAT box motifs in c-Fos(only, distal, AP-1−) regions. Instead an alternative AP-1 motif was found to be enriched in HeLa S3 and K562 intervals (Fig 3).

In this context we analyzed the co-localization pattern related to enhancer and promoter regions for the transcription factor c-Jun. The ENCODE project offers three different c-Jun ChIP-seq experiments that are overlapping with cell lines used in this study: (i) HUVEC, (ii) HeLa S3, and (iii) K562. For the enhancer related genomic intervals that are characterized by at least one AP-1 motif we found 52% (14551 intervals), 68% (2360 intervals), and 95% (1434 intervals) which co-localize with c-Jun precipitated genomic intervals for these three cell lines. We conclude that these regions are mainly regulated by AP-1 and that the AP-1 transcription factor is assembled through the c-Fos/c-Jun heterodimer complex. Interestingly, we found no equivalent co-localization pattern for the corresponding promoter sets. Only 38% (467 intervals), 10% (68 intervals), and 15% (288 intervals) of promoter related c-Fos precipitated ChIP-seq intervals are overlapping with c-Jun intervals for the HUVEC, HeLa S3, and K562 cell lines. Summarizing these observations we suppose that c-Fos is the main interaction partner for our proposed enhancer/promoter model. As a member of the enhancer bound AP-1 heterodimer it is interacting with the transcription factor NF-Y (directly or indirectly) that is bound to the CCAAT box or CCAAT box repeats in their corresponding promoter regions.

Our data analysis thus demonstrates that proximal c-Fos precipitated ChIP-seq intervals that are not co-localized with NF-Y related binding events contain CCAAT motifs and at the same time were lacking AP-1 motifs. This observation was found in all three cell-types for proximal ChIP-seq intervals.

### CCAAT and AP-1 motifs are mutually exclusive in c-Fos-targeted regions

To get a more robust criterion for the co-occurrence probability of these two regulatory elements, we checked the appearance of high-scoring potential AP-1 and NF-Y binding sites in the proximal and distal regions bound by c-Fos in either of the four cell lines. We recorded the presence of AP-1 or NF-Y binding motifs using TRANSFAC/Match [20, 22] and applying a threshold that was optimized with regard to Matthews Correlation Coefficient (MCC) (see Methods and S1 and S2 Figs, bottom insets).

As can be seen in Table 4, by far most of the c-Fos-targeted regions in HUVEC cells only possess an AP-1 motif, and very few exhibit an NF-Y binding motif. The opposite is true for the GM12878 cell line: It has very few c-Fos-bound distal regions (290 compared with more than 20,000 in HUVEC cells), and is practically lacking AP-1 motifs in either type of regions. It is also noticeable that considerable portions of the c-Fos-targeted regions exhibit neither a high-scoring AP-1 nor NF-Y binding motif (between 15.2% in HeLa S3 proximal regions and 46.2% in HUVEC proximal regions). These intervals will be investigated in a separate study.

**Table 4:** High-scoring potential binding sites for AP-1 and NF-Y appear in a mutually exclusive way. The column AP-1 gives the number of genomic intervals bound by c-Fos in the indicated cell line, which have at least one match with the AP-1-related matrix V$JUNDM2_04 exceeding the optimized MSS and do not exhibit an NF-Y match as well. Correspondingly, the column NF-Y gives the number of genomic intervals matching with the matrix V$NFY_01 without any AP-1 motif match above the threshold. Column both gives the number of double occurrences, the column no AP-1/NF-Y the number of intervals with neither motif. Proximal (p) and distal (d) regions are shown separately.The Odds, Odds ratio and the Fisher’s Exact p-Values are calculated based on the cell type specific proximal and distal group in relation to an AP-1 or NF-Y binding site prediction.

Cell line	type	AP-1	NF-Y	Odds	Odds Ratio(P-value)	both	AP-1−,NF-Y−	sum
HUVEC	p	500	98	5.102	0.034	64	569	1231
	d	14270	95	150.211	(7.811e-86)	281	5535	20181
HeLa S3	p	86	451	0.191	0.002	20	100	657
	d	3661	38	88.447	(0)	60	696	4455
K562	p	79	1392	0.0568	0.021	32	409	1912
	d	1536	574	2.676	(0)	91	448	2649
GM12878	p	4	989	0.004	0.029	27	265	1285
	d	18	130	0.138	1.935e-13	15	127	290

**Fig 4:**
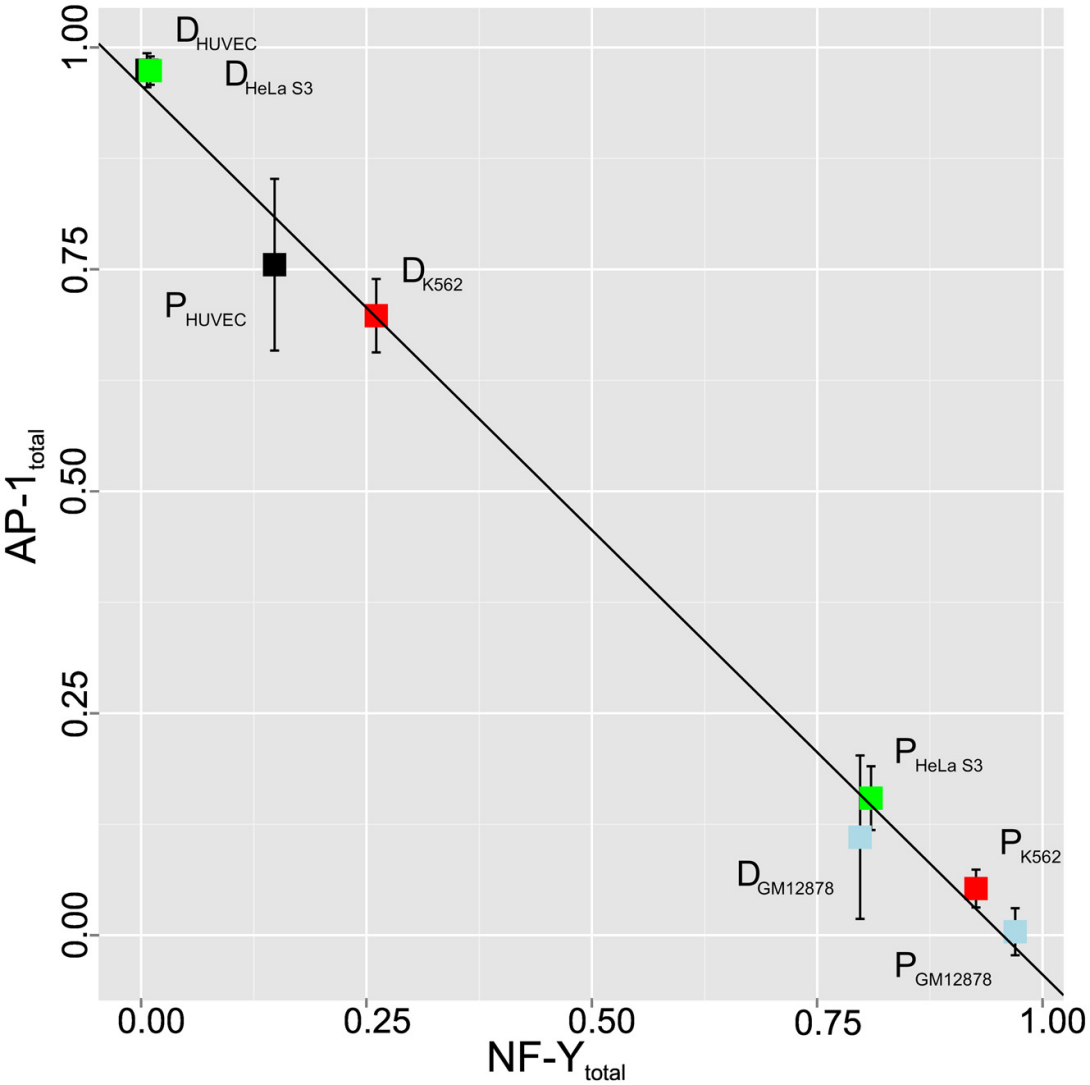
Anti-correlation of the occurrence of AP-1 and NF-Y binding motifs. The percentage of genomic intervals that harbor an AP-1 motif was calculated and plotted against the percentage of those harbboring an NF-Y. P_X_ are proximal, D_X_ distal intervals from cell line X. Note that two sequence sets HUVEC distal (D_HUVEC_, black color) and HeLa S3 distal (D_HeLa S3_, green color) concur with the left-most point.

Focusing on those c-Fos-bound ChIP-seq interval sets that exhibit either motif, we observed in the sequence sets shown in Table 4 that the higher the percentage of AP-1 motif occurrence is, the lower is that of the NF-Y binding motifs and vice versa. Plotting these values against each other for all eight sequence sets, we found a strict linear anti-correlation (*R* = −0.9571; Fig 4). This plot shows a big gap between putative AP-1 and NF-Y regulated c-Fos ChIP-seq regions. The appearance of high-affinity binding motifs of AP-1 and NF-Y appear in a mutually exclusive way.

**Fig 5:**
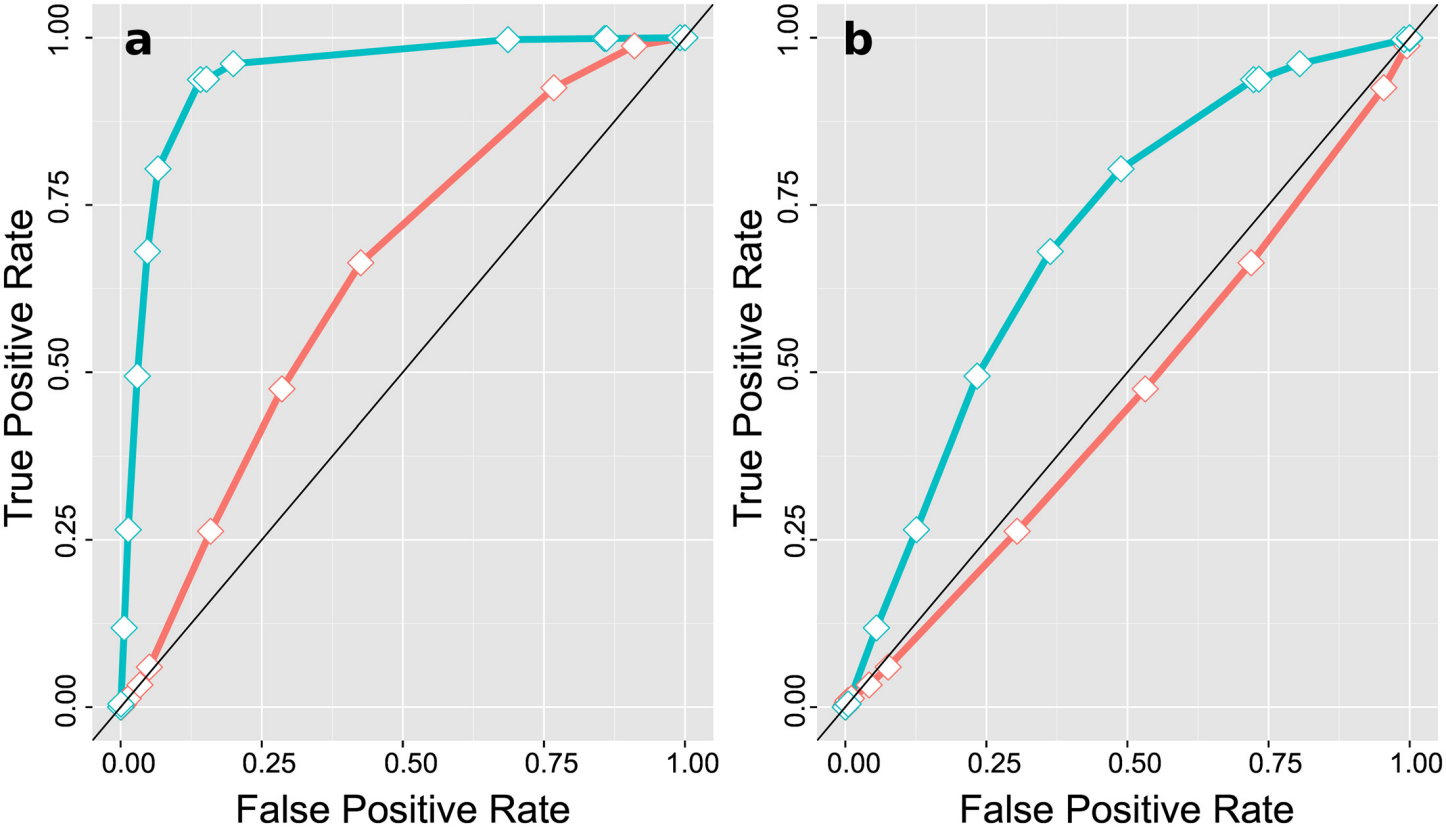
ROC curves for AP-1 and NF-Y binding motifs for co-localized c-Fos and NF-YB intervals. The ROC curves were monitored using TRANSFAC matrix V$JUNDM2_04 for the AP-1 motif (red) and V$NFY_01 for the NF-Y binding motif (blue).

We monitored the ROC curves for both motifs in the union set of all proximal intervals where c-Fos and NF-YB co-localize in at least one of the three cell lines (HeLa S3, K562, and GM12878) compared to non-overlapping DHS sites as background set (control set). In Fig 5(a), it is shown that these proximal co-localized regions are enriched for high-affinity NF-Y motifs (blue curve) and at the same time are depleted high-scoring (related to the MCC) AP-1 motif predictions (red curve, left bottom). When we replace the DHS background set by NF-YB(only) ChIP-seq regions, the CCAAT motif shows identical behavior in both data sets (ROC curve at the diagonal). In this case, the AP-1 ROC curve is even slightly below the diagonal, indicating a slight deprivation of AP-1 motifs in the c-Fos/NF-YB co-localization set (Fig 5(b)).

Based on the observed result we conclude a mutually exclusive behavior for c-Fos-targeted regions if they are containing high-affinity AP-1 or NF-Y motifs. Enhancer regions active in HeLa S3 or in K562 cells were characterized by AP-1 motifs, promoters of these two cells were enriched for CCAAT box motifs. The GM12878 promoters are shaped by CCAAT boxes as well, but due to the low number of c-Fos-bound enhancers in these cells no statistical statement can be made upon their motif composition.

### Many c-Fos-bound genomic intervals exhibit CCAAT dimers in a specific configuration

As was observed from the MEME analyses, c-Fos-targeted proximal regions tend to exhibit two distinguishable variants of CCAAT boxes (S4–S6 Figs, left part). They show slightly different preferences in their flanking regions to the otherwise perfectly conserved CCAAT box. For the following investigations, we therefore restricted our search to the CCAAT element as a regular expression. Moreover, to improve the statistical basis for the further experiments, we united the sequence sets for the three cell lines HeLa S3, K562 and GM12878. We put into the intersection all those regions, either proximal or distal, where transcription factors c-Fos and NF-YB co-localize in at least one of these cell lines. This group is called Inter(NF-YB, c-Fos). The c-Fos(only) group is made out of ChIP-seq intervals which are found to interact with c-Fos and had no overlap to any NF-YB bound region of the three cell lines under study. The NF-YB(only) group defined analogously for NF-YB ChIP-seq regions only.

**Fig 6:**
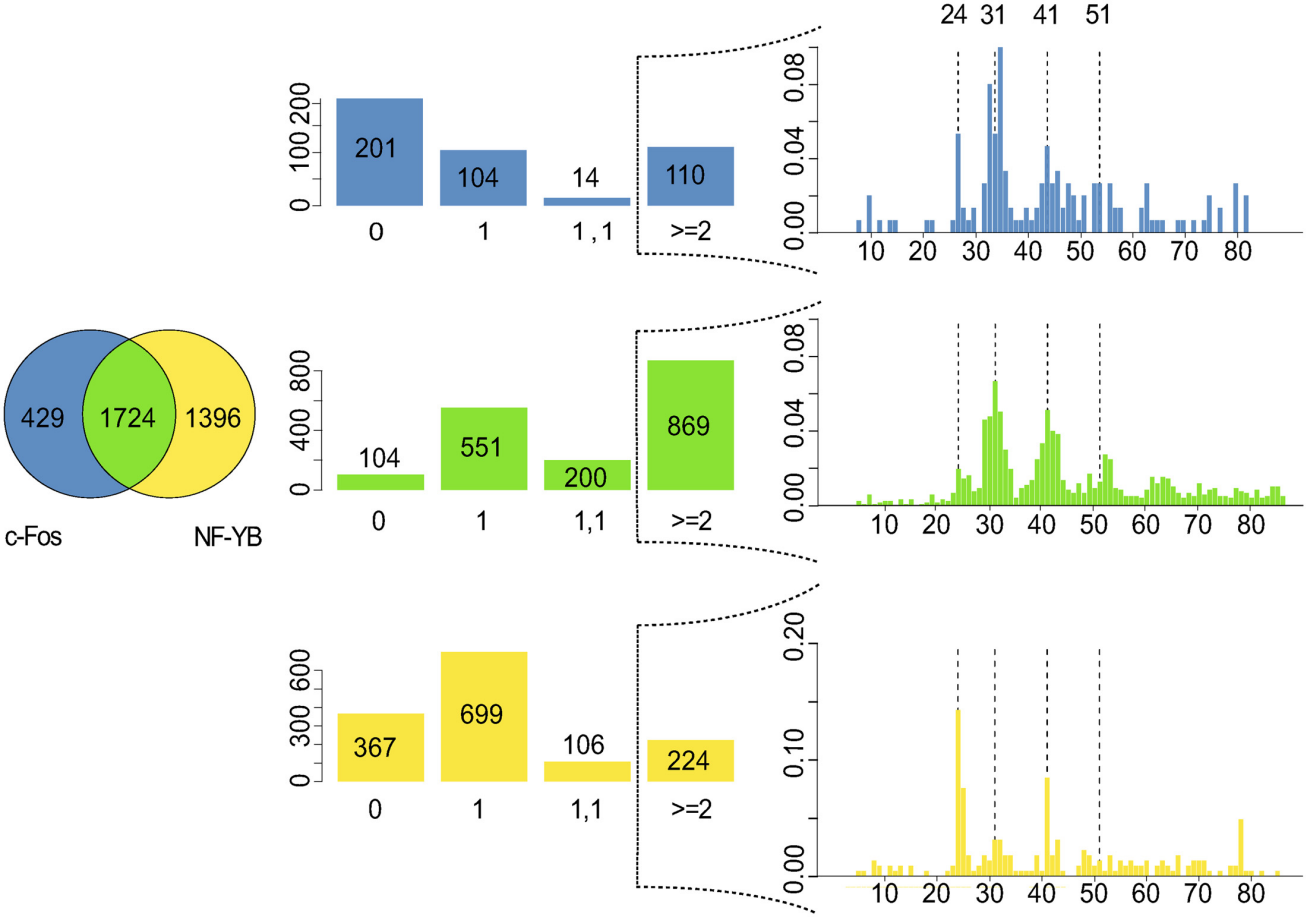
Distribution of CCAAT boxes in c-Fos-bound proximal regions. Shown are the numbers of ChIP-seq genomic intervals found in three cell lines (HeLa S3, K562, GM12878) that interact either with c-Fos (blue) or NF-YB (yellow), or with both of them (green). Left: Venn diagram displaying the distribution of proximal intervals among the three classes. Center: Histograms showing the numbers of intervals having no CCAAT box motif (0), one CCAAT box (1), two CCAAT boxes as inverted or everted repeat (1,1), or two or more CCAAT boxes as direct repeat (> = 2) related to the three data sets. The exact frequencies are given in each column. The histograms on the right show the distance distribution of the direct repeats for each class. The dashed lines highlight the distances 24 bp, 31 bp, 41 bp, and 51 bp.

**Fig 7:**
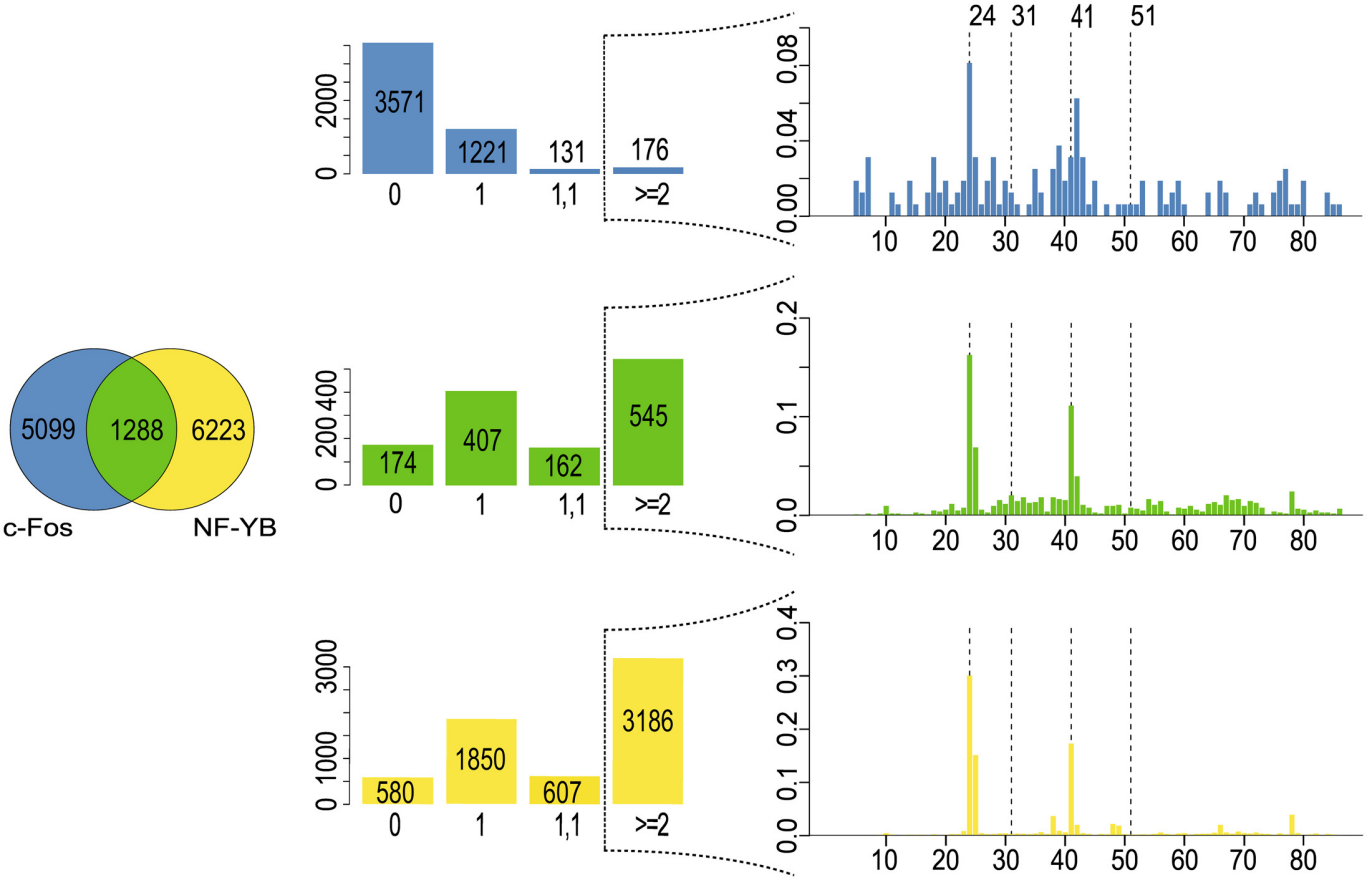
Distribution of CCAAT boxes in c-Fos-bound distal regions. Shown are the numbers of ChIP-seq intervals found in three cell lines (HeLa S3, K562, GM12878) that interact with c-Fos (blue) or NF-YB (yellow), or with both of them (green). Left: Venn diagram displaying the distribution of proximal intervals among the three classes. Center: Histograms showing the numbers of intervals having no CCAAT box motif (0), one CCAAT box (1), two CCAAT boxes as inverted or everted repeat (1,1), or two or more CCAAT boxes as direct repeat (> = 2). The histograms on the right show the distance distribution of the direct repeats for each class. The dashed lines highlight the distances 24bp, 31bp, 41bp, and 51 bp.

The Figs 6 and 7 show the numbers for these three sequence sets as Venn diagrams, separately for proximal (Fig 6, left) and distal regions (Fig 7, left). The central histograms indicate how many intervals harbor no CCAAT box that would be recognizable as regular expression (column 0), or possess a single CCAAT box (column 1), or two CCAAT boxes as inverted or everted repeat at any distance (column 1, 1), or at least one tandem repeat of two neighboring CCAAT boxes at any distance (column > = 2) separated for proximal or distal intervals (Fig 6, center; Fig 7, center). The observed frequencies are also shown in each bar of the corresponding histograms. The results illustrate that the occurrence of direct repeats was much higher compared to the frequencies of in-/everted repeats. The only exception was found for the c-Fos(only) distal regions (Fig 7, center, top histogram). The majority of these intervals did not contain a CCAAT box at all, but were rather characterized by classical AP-1 motifs.

**Fig 8:**
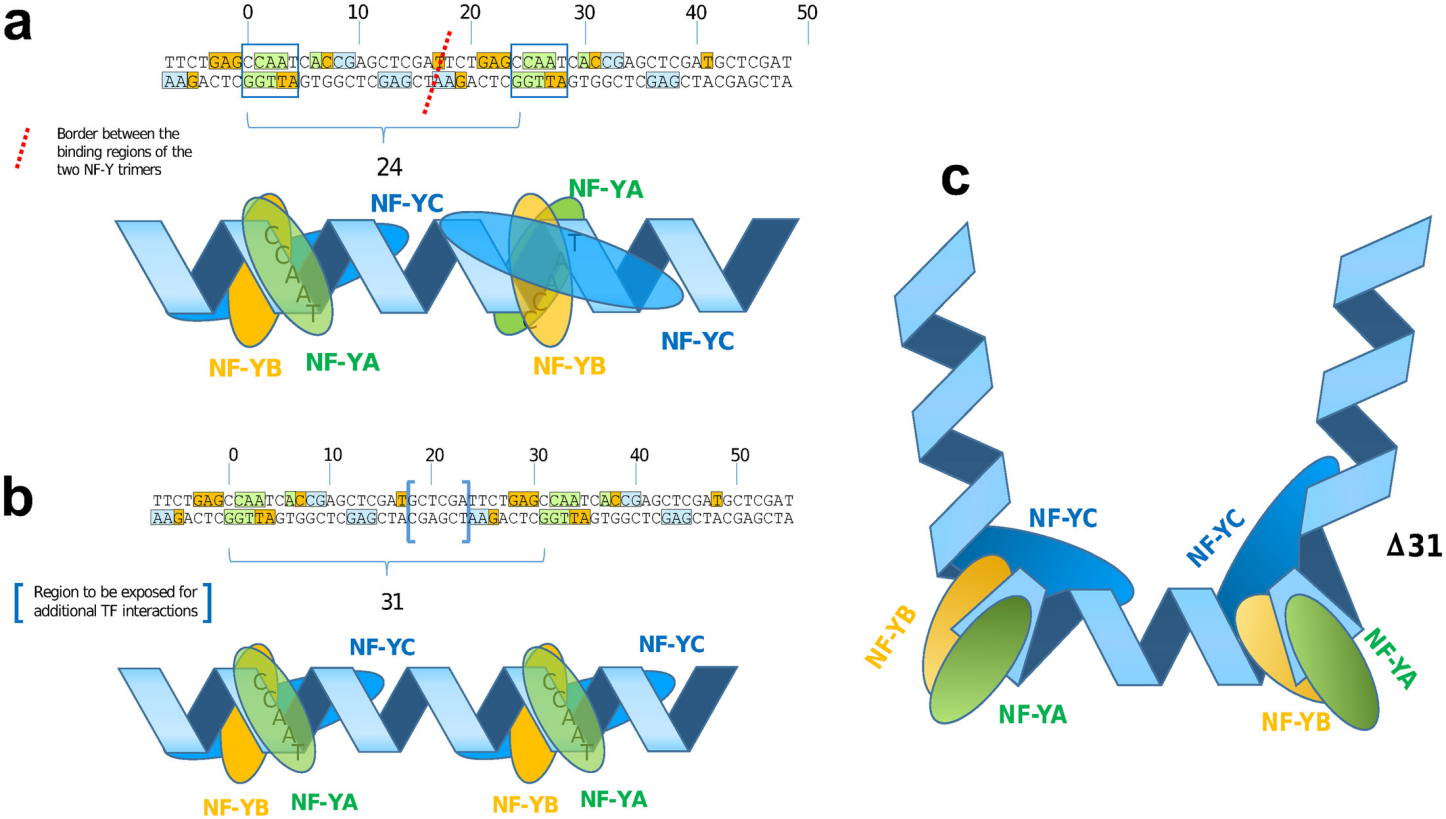
CCAAT box direct repeats confer NF-Y binding sites that are immediately adjacent or exhibit a small gap. (**a**) LTR shaped CCAAT box direct repeat model which can frequently found in distal regions. (**b**) CCAAT dimer model of c-Fos-targeted promoters. (**c**) Proposed binding model of proximal CCAAT box direct repeat.

The direct repeat situation for the three groups c-Fos(only), Inter(NF-YB, c-Fos), and NF-YB(only) has been studied in more detail. The Fig 6 (right) and Fig 8 (right) summarize the observations for the proximal and distal sequence sets. We analyzed the distance distribution of neighboring CCAAT boxes in a tandem arrangement (see Methods for details). The most pronounced feature of c-Fos-bound proximal regions, independently of whether they appear in c-Fos(only) or Inter(NF-YB, c-Fos) group, is a peak around a distance value of 31bp, accompanied by a series of progressively decreasing maxima at every 10-11bp in addition. Two peaks that appear at distances 24 and 41 sharply become most prominent in the group of distal regions bound by NF-YB(only). This characteristic profile can be found in the distal Inter(NF-YB, c-Fos) regions as well. Closer inspection revealed that they correspond to a specific repetitive element class, the so-called Long Terminal Repeats (LTR), in particular the LTR12 family. Their appearance among the c-Fos-bound proximal regions may be considered as “contamination” of this promoter class by these LTR sequences, which are known to interact with the transcription factor NF-Y [18].

We conclude that c-Fos-targeted promoters that do not exhibit an AP-1 motif but are characterized by direct CCAAT box repeats, preferable at a distance of 31bp, define a novel sequence-specific regulatory module bound by the transcription factor NF-Y.

### AP-1 positive enhancers tend to be close to c-Fos-targeted promoters

It has been discussed earlier, that enhancer-bound proteins may be cross-linked by the ChIP procedure also to promoters targeted by these enhancers [12]. We therefore decided to investigate whether c-Fos-bound enhancer, characterized by AP-1 motifs, relate to c-Fos and NF-YB bound promoters with a CCAAT direct repeat. For this, we analyzed the distal and proximal data for putative enhancer/promoter distance distribution. As potential promoters we took the 1724 proximal regions which are targeted by c-Fos as well as by NF-YB (Fig 6, Venn diagram). Two groups of putative enhancers were defined as follows: As the first enhancer set we used c-Fos-targeted distal intervals that exhibit no CCAAT motif, but were found to be highly enriched in high-scoring AP-1 motifs (in total 3571 segments, see Fig 7). The second enhancer set (control) was defined by NF-YB targeted ChIP-seq intervals with at least two CCAAT motifs in a direct tandem arrangement, which are mostly LTR elements (in total 3186 segments, see Fig 7). Either group was sampled 1000 times into equal-sized subgroups of 1500 sequences, and the distance distribution from each promoter to the closest enhancer of the one or the other group was recorded (see Methods for details). The distance distributions are plotted in S8 Fig and show that the c-Fos-bound and AP-1 motif shaped enhancers show a clear tendency to be closer to c-Fos-bound and CCAAT box characterized promoters than the control distal regions (Mann-Whitney Test: *P* = 3.31703*e* − 281).

The ENCODE project investigated a genome-wide annotation of chromatin state for different cell lines [23]. Based on these data we verified our cell type specific distal (enhancer) and proximal (promoter) annotations. The following cell lines related to our study are available: HUVEC, K562, GM12878. We investigated the cell type specific overlaps of our double CCAAT associated data sets for promoter specific chromatin modifications. Three different proximal data sets were used: (i) Proximal c-Fos and NF-YB co-localized genomic intervals (Inter). (ii) Filtering of the Inter set based on the double CCAAT as direct repeat (Inter(CCAAT2+)). (iii) Co-localization of the Inter(CCAAT2+) group with p300 interacting regions. The Table 5 is summarizing the results.

**Table 5:** Chromatin state of proximal genomic intervals. We used cell type specific chromatin state data (Broad ChromHMM) [23] to identify the proportion of the proximal genomic intervals in relation to ENCODE defined promoters. We analyzed the overlaps for the HUVEC, K562, and GM12878 cell lines (columns).

Proximal data sets	HUVEC	K562	GM12878
Inter [1724]	1535 (89%)	1543 (90%)	1557 (90%)
Inter(CCAAT2+) [869]	773 (89%)	770 (89%)	791 (91%)
Inter(CCAAT2+, p300) [471]	424 (90%)	426 (90%)	438 (93%)

For all three data sets (i-iii) we observe a large overlap (89–93%) to chromatin modifications that are observed for active promoter regions. This result verifies our proximal data sets as promoter regions and support the relevance of the double CCAAT box as a main feature in this data sets.

To verify our distal genomic intervals as potential enhancers we determine the overlap of the distal genomic intervals to chromatin modifications related to active enhancers. The Table 6 is summarizing this situation.

**Table 6:** Chromatin state of distal genomic intervals. We used cell type specific chromatin state data (Broad ChromHMM) [23] to identify the proportion of the high-scoring AP-1 motif containing distal genomic intervals that overlap with ENCODE defined enhancers. We analyzed the overlaps for the HUVEC, K562, and GM12878 cell lines (rows) and relate them to the AP-1+ motif containing genomic intervals.

Cell line	AP-1+ (distal)	Broad ChromHMM
HUVEC	14551	11307 (78%)
K562	1505	1340 (90%)
GM12878	8	5 (64%)

AP-1 motifs that are characterizing distal genomic intervals show a large overlap with active enhancer regions defined by the ChromHMM data sets.

Two-thirds of all enhancer/promoter interactions in the GM12878 cell line are directed to the nearest active promoter [24]. Based on our results and the aforementioned statement we propose a new functional coupling between AP-1 bound enhancer regions and their corresponding proximal regions shaped by these NF-Y bound CCAAT dimers.

### c-Fos-targeted regulatory regions also interact with co-activator p300

Both transcription factors c-Fos as well as NF-Y are known to interact with the co-activator p300 by binding either to the C-terminal or a central region of the p300 molecule, respectively [25, 26]. Since most of the c-Fos-targeted promoters studied here are bound by NF-Y via CCAAT boxes, it was tempting to hypothesize that binding of c-Fos to these promoters might be mediated by p300. Indeed, we observed a strong co-localization pattern of c-Fos ChIP-seq regions with genomic intervals bound by the co-activator p300. 87% of all distal c-Fos-targeted regions are bound by p300 (Table 7). Only 65% of the proximal c-Fos-precipitated regions also interact with p300. However, when we split the proximal set in AP-1(+) and AP-1(−) subsets depending on the presence/absence of a high-scoring AP-1 motif, we noticed that the former overlapped to 83% with the p300 ChIP-seq set, while only 57% of the AP-1(−) intervals exhibited a detectable p300 overlap. It is interesting to note that the two AP-1(+) data sets, c-Fos(only, distal) and c-Fos(only, proximal) filtered for high-scoring AP-1 (AP-1+) show very similar large overlap with p300 ChIP-seq intervals (87% and 83%, resp.). The CCAAT shaped proximal interval groups c-Fos(only, AP-1−), Inter(NF-YB, c-Fos), and NF-YB(only) show a moderate but comparable p300 overlap as well (57%, 57%, 56%). Much less co-localization has been observed for CCAAT characterized distal regions.

**Table 7:** Distal and proximal p300 overlap of unified c-Fos and NF-YB ChIP-seq regions. The total numbers of ChIP-seq intervals that are bound either by c-Fos, NF-YB, or by both of them are the same as in Figs 6 and 7. The column p300 overlap depicts the number of them that overlap with p300 ChIP-seq intervals, expressed as percent in the last column.

Region	Class	Total	p300 overlap	Percentage
distal	c-Fos(only)	5099	4436	87%
	Inter	1288	556	43%
	NF-YB(only)	6223	1335	22%
proximal	c-Fos(only)	429	279	65% (AP-1+ 83%, AP-1− 57%)
	Inter	1724	979	57%
	NF-YB(only)	1396	786	56%

### Functional annotation of proximal c-Fos and NF-YB promoter regions

In order to detect potential functional correlates of the c-Fos(CCAAT) promoters, in particular those that harbor a direct CCAAT repeat, we mapped all proximal regions into three classes: c-Fos(only), Inter(NF-YB, c-Fos), or NF-YB(only) (see Fig 6). From the regions of the Inter group (1724 intervals) we selected those that contain a direct dimeric repeat of CCAAT boxes and labeled this group Inter(CCAAT2+). The group Inter(CCAAT2−) contains all intervals that are void of such a repeat. Both groups are of about about equal size (869 and 855 intervals, resp.). Using RefSeq annotation (see methods for details) we are able to retrieve 634 unique gene symbols for the Inter(NF-YB, c-Fos) group. The Inter(CCAAT2+) set is made out of 344 genes and for the Inter(CCAAT2+, p300) we retrieved 184 unique gene symbols. We subjected these groups of intervals to a gene ontology (GO) term enrichment analysis using the GREAT tool [27].

For the c-Fos(only) set, we did not find any enrichment of a GO-defined biological process [28] after p-value correction, and for the NF-YB(only) data set, we observed some very general biological process categories to be enriched (data not shown). For the Inter group, we found that most of the top enriched terms relate to nucleosome and chromatin organization (S2 Table). This trend was even more pronounced when we analyzed the two subgroups of this class. While the Inter(CCAAT2−) subset, like the NF-YB(only) group characterized above, just exhibited some very generic categories to be enriched, the subset Inter(CCAAT2+) showed very significant over representation of chromatin/nucleosome assembly and related processes (S3 Table). When we focused on those intervals of this group that also overlap with p300 binding regions this enrichment became even more pronounced (S4 Table). In Table 8, we show the progressive enrichment of these GO categories when proceeding through these three filtering steps; sorting of categories was done according to hyper-geometric Q values (FDR corrected p-values), obtained after the last filtering step. Shown are all 11 GO categories that appear among the top 20 ranking terms in all three data sets. Interestingly, and suitable for an internal control, among these 20 GO terms are three that do not belong to the mentioned group. In contrast to the genes acting in nucleosome and chromatin organization, those belonging to any of these three categories are depleted during the described selection.

**Table 8:** GO categories enriched among genes targeted by c-Fos and NF-YB. We observed a progressive enrichment of a set of GO categories represented in this table, when proceeding three filtering steps. The Inter group is made out of 1724 proximal intervals, 869 intervals of this group contain a least a CCAAT box direct repeat. The last consists of 470 proximal ChIP-seq intervals (Inter(CCAAT2+,p300)). The top 20 enriched categories for each data set can be found in S2–S4 Tables.

Gene Ontology category (#total)	Inter (# genes)	Inter(CCAAT2+) (# genes)	Inter(CCAAT2+,p300) (# genes)
nucleosome assembly (144)	8.3661e-23 (66)	1.0935e-30 (59)	9.6057e-32 (48)
protein-DNA complex assembly (166)	2.0001e-22 (71)	6.4928e-30 (62)	4.5491e-31 (50)
chromatin assembly (156)	3.1488e-21 (67)	6.9986e-30 (60)	2.2627e-30 (48)
nucleosome organization (167)	4.3924e-20 (68)	3.3856e-27 (59)	5.8612e-29 (48)
DNA conformation change (232)	1.4360e-18 (80)	1.2123e-27 (70)	7.3449e-29 (55)
protein-DNA complex subunit organization (189)	4.6583e-20 (73)	9.6572e-27 (62)	1.5355e-28 (50)
DNA packaging (193)	6.6237e-17 (69)	3.2159-e26 (62)	3.4329e-28 (50)
chromatin assembly or disassembly (175)	7.2675e-19 (68)	6.0315e-27 (60)	3.6575e-28 (48)
response to endoplasmic reticulum stress (127)	3.4353e-07 (39)	7.1109e-05 (24)	0.0007 (16)
positive regulation of nuclease activity (67)	1.3394e-07 (27)	0.0010 (15)	0.0302 (9)
regulation of nuclease activity (73)	2.4459e-07 (28)	0.0025 (15)	0.0497 (9)

The explicit enrichment of chromatin and nucleosome assembly process categories in proximal regions of nearby genes, where (a) c-Fos- and NF-YB-co-localize, (b) the direct CCAAT tandem arrangement is present, and (c) co-activator p300 co-localizes may indicates that direct CCAAT boxes have a distinct regulatory influence on these processes. These repeats are the dominating feature in this group and contain no high-scoring AP-1 motifs. We hypothesize that these dimers define an appropriate interface for a transcription factor complex which comprises c-Fos, NF-YB and, probably, the co-activator p300.

## Discussion

### Relation between CCAAT boxes and AP-1 motifs

During our analysis of the different c-Fos related ChIP-seq data sets, we made a couple of noteworthy observations. The first was that many c-Fos-targeted promoters exhibit an AP-1 motif, as to be expected, but that an even higher number is rather characterized by a CCAAT box; since these promoters are also found in an NF-YB data set, their CCAAT boxes serve obviously as NF-Y binding sites. This finding confirmed earlier observations on the same data sets, which have already been described in factorbook [29] as being predominantly characterized by NF-Y binding motifs (http://www.factorbook.org/mediawiki/index.php/C-Fos). Also, Fleming et al. reported co-localization of NF-Y and c-Fos at CCAAT, typically at loci lacking an AP-1 motif [18]. One explanation could be that c-Fos, either alone or as AP-1 heterodimer with a Jun-related component, binds to the DNA-bound NF-Y through protein-protein interactions. Coss et al. reported co-binding of AP-1 and NF-Y to the promoter of the gonadotropin-releasing hormone (GnRH) gene, and that this is achieved by a direct interaction between c-Jun and NF-YA [30]. Co-immunoprecipitation of c-Fos and NF-YB was shown by Xie et al. [11]. However, such an interaction alone would not explain why some promoters containing a CCAAT-box are targeted by c-Fos but most are not, although all are bound by NF-Y, as evidenced by NF-YB ChIP [18]. The reason for this differential activity must be encoded in the genome. The most trivial case would be the existence of low-affinity, degenerate AP-1 motifs in the environment of c-Fos(CCAAT) promoters. The presence of such AP-1 motifs has not been supported by the ROC curves shown here, which in such a case should show a forced increase in the higher FPR range, nor have we been able to show enriched low-scoring AP-1 motifs compared with the NF-YB sequences that are not targeted by c-Fos (data not shown). Also Fleming et al. [18] have not found AP-1 motifs in the neighborhood of c-Fos-targeted NF-Y bound regions, with the exception of LTRs (see below). This is explicitly also true for potential AP-1 sites overlapping with the NF-Y consensus, which has some tendency to form an AP-1 half-site motif (TGA or TCA) at its 3’-end (underlined): CCAAT(c/g)a. A particular instance with a potential AP-1 half site 5’ to the CCAAT box was suggested as well [30]. However, our studies did not reveal any significant difference in the immediate flanks of the CCAAT boxes located in c-Fos-targeted or -non-targeted promoters. The same was true for other accompanying motifs. We consistently found the well-known Sp1 binding motif in the environment of the CCAAT boxes, but this also was to be observed independently of the c-Fos-targeting of the respective promoters. No accompanying motif was found that could be a candidate discriminator between the two sets of CCAAT box promoters. The lack of recognizable AP-1 motifs in c-Fos(CCAAT) promoters is a clear evidence against a similar co-localization mechanism between NF-Y and AP-1 sites as it is known for NF-Y and Sp1 binding [31].

Moreover, our second observation was that CCAAT boxes and AP-1 motifs even appear in a mutually exclusive way. If a high-scoring AP-1 binding site is found in a c-Fos ChIP-seq region, no NF-Y binding motif is detectable and vice versa by motif matching (TRANSFAC/MATCH) or motif discovery (MEME) approaches. We conclude that c-Fos-targeting of promoters that are characterized by CCAAT boxes even depends on the absence of a genuine AP-1 binding binding site in the same promoter. We suppose an interfering mechanism of such an AP-1 binding motif which prohibits formation of a proper enhanceosome.

### Structural implications of the CCAAT box dimers

While we could exclude the presence of obscure AP-1 motifs or other accompanying binding sites to explain the c-Fos-targeting to c-Fos(CCAAT) promoters, we checked whether any particular arrangement of the CCAAT boxes could provide an explanation. We were able to identify recurring patterns of two tandem copies of CCAAT boxes specifically in the c-Fos-targeted promoters, with a pronounced distance distribution profile. The main peak is at a distance of 31 base pairs between the two CCAAT boxes, followed by decreasing maxima of around 41, 52 and 63 bp. The difference of 10-11bp between these peaks indicates a conserved orientation of the two CCAAT boxes on the same side of the DNA double helix. It has been shown by structural analysis of NF-Y/DNA co-crystals that the NF-Y heterotrimer induces a strong bending of the DNA by about 80 degree between the second A and the T of the CCAAT box [32]. Since the distance between the two CCAAT boxes preferably is an integer multiple of one complete DNA turn, the two kinks point into the same direction resulting in a slightly diverging U-like structure (Fig 8(c)). Shorter distances than three turns would not be feasible since subunit NF-YB contacts reach out to position +17 (starting with the first C of the upstream CCAAT box as position 0) and subunit NF-YC has contacts up to position -7. The minimum distance between two NF-Y binding sites is therefore 24 base pairs. This corresponds exactly to the most prominent peak found in the CCAAT dimer distance distribution in NF-YB-bound distal regions (Fig 8(a)), arising from repetitive elements of LTR-type 12, as we and others have found [18]. In these CCAAT dimers with distance 24bp, both NF-Y trimers bind immediately adjacent to each other, with the kinks two and a half turns apart causing a more S-like structure. In contrast, the U-shaped NF-Y bound CCAAT dimer at distance 31 bp may expose a short stretch of DNA between the two NF-Y binding areas, which may be targeted by other TFs (Fig 8(b)).

### The role of enhancers

**Fig 9:**
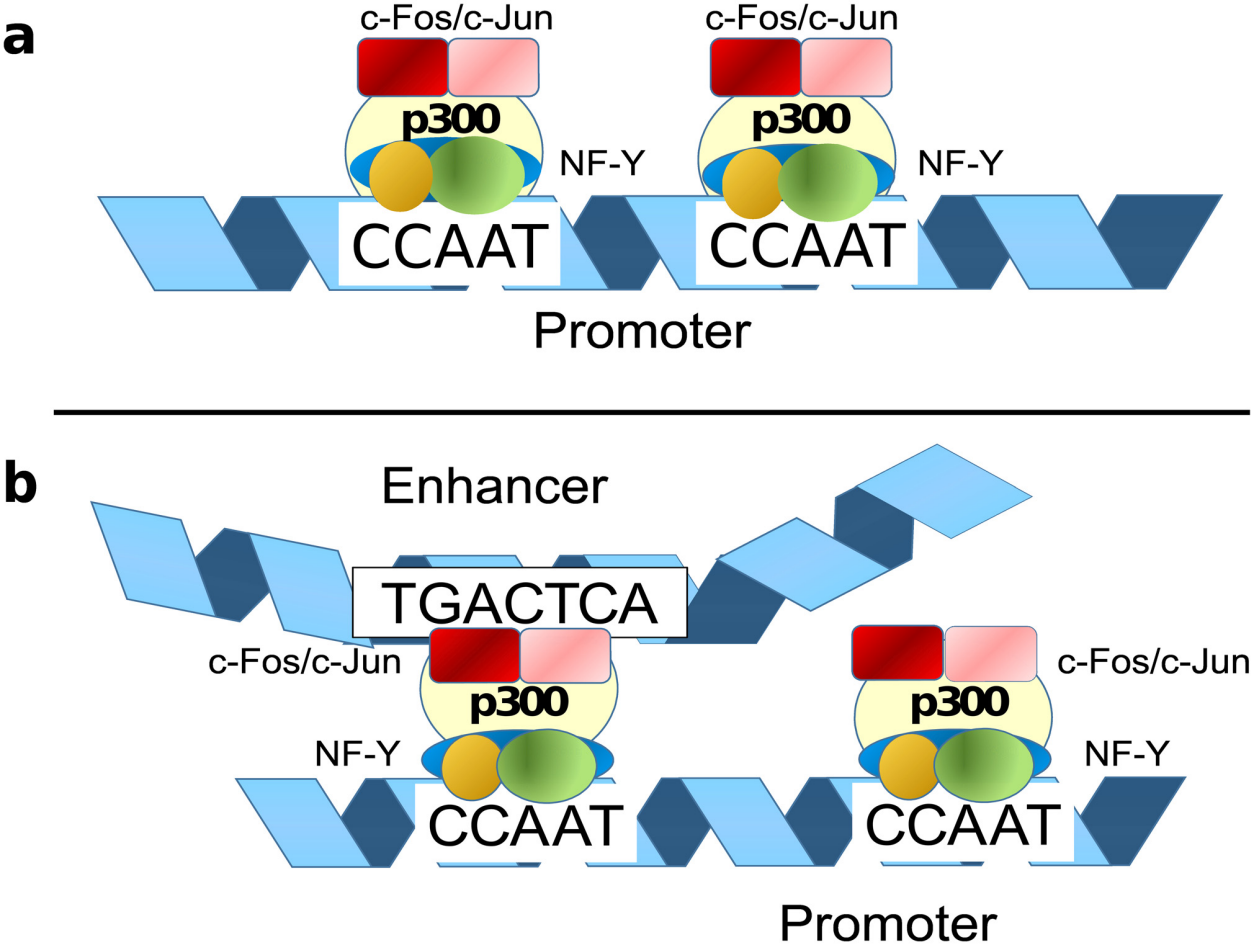
Hypothetical models of c-Fos/NF-YB interactions. The first model (**a**) describes our general understanding of the interaction between the TF c-Fos and the CCAAT box conferring NF-Y binding. (**b**) Alternative interaction model in which AP-1 is contributed to the whole enhanceosome by the enhancer, targeting the promoter (CCAAT) bound NF-Y via the co-activator p300.

In contrast to the promoters related intervals, most of the enhancer intervals that are targeted by c-Fos in the cell lines HeLa S3 and K562 exhibit a clear AP-1 motif. This prompted the speculation that it might have been the enhancers that contribute to the c-Fos-binding to the respective promoters, detected by ChIP after cross-linking of protein complexes that were bound to both enhancers and promoters as discussed by Mercer and Mattick [12]. This was supported by our finding that c-Fos(AP-1+) enhancers tend to be closer to c-Fos(CCAAT) promoters, to which they were supposed to exert physical contacts, than to NF-YB(CCAAT) promoters that are not targeted by c-Fos (see Fig 9). Such a contact could either be a direct one, for instance via the above-mentioned potential interactions between c-Jun and NF-YA [30] or c-Fos and NF-YB [11]. In this study we have shown that c-Fos and at the same time AP-1 motif containing enhancers (AP-1+) show a significant co-localization pattern with c-Jun precipitated ChIP-seq intervals. Alternatively, such a contact could be mediated by a co-activator such as p300. This co-activator is known to interact with both TFs, c-Fos [25, 33] and NF-Y [26], through different binding sites: c-Fos is bound to a C-terminal part of the p300 molecule (positions 1572–1818), where also the adenoviral TF E1A binds [25]. NF-Y interacts with a mid-regional part of p300, positions 671–1194 [26], encompassing a bromo domain (1067–1139) and a cell-cycle regulatory domain CRD1 (1017–1029; UniProt: Q09472). As a consequence, both factors AP-1 and NF-Y can simultaneously bind to p300 [34]. Interestingly, significant portions of c-Fos-targeted proximal and distal regions are also bound by p300. This proportion is by far the lowest for distal regions that are bound by NF-YB, but are not targeted by c-Fos (22%). In contrast, it is the highest for c-Fos-targeted distal regions, most of them with a recognizable AP-1 motif (87%) and to the few c-Fos-targeted promoters that have an AP-1 motif (83%), which we actually suspect to have been misclassified as promoters because of their vicinity to a TSS. We summarize these findings by proposing a model where the co-activator p300 is mainly recruited by enhancer-bound c-Fos/AP-1, which then loops back to the promoter where it contacts the two NF-Y complexes bound to a CCAAT direct dimeric repeat, preferably with a 31bp distance between the two CCAAT boxes (see Fig 9). This model may work for those c-Fos-targeted genes that were identified in Hela S3 and in K562 cells. For some reason, such genes in GM12878 cells are regulated by c-Fos through a different mechanism. Here, practically no c-Fos-targeted enhancers are found which could recruit p300. Instead, we speculate that in these cases, c-Fos (or AP-1) already complexed with the co-activator binds to NF-Y/CCAAT dimer promoters without being supported by an enhancer (see Fig 9). The reason for this different behavior remains unclear so far, but may reflect a particular cellular condition in this cell line.

### Functional implications

The functional categorization of genes with c-Fos-targeted promoters revealed as most prominent GO classes those that relate to chromatin remodeling processes. One of the most prominent functions of the FOS proto-oncogene is to promote cell proliferation, but it also plays a role in other processes like cell differentiation mediating some signal responses. On the other side, NF-Y has been suggested to be a master regulator of cell cycle progression, recruiting p300 to cell cycle regulatory genes [35]. As mentioned above, it does so through a cell cycle regulatory domain of p300 [26]. It may therefore be tempting to speculate in the light of the findings reported here, that at least a certain subset of such genes is tagged by NF-Y bound to a CCAAT dimer and induced by c-Fos and AP-1 recruited p300. The CCAAT dimer with a distance of 31bp would thus match the characteristics of a typical seed site, as we have defined earlier [36].

### Biological implications of a CCAAT box direct repeat

In a study from Lok et al. [37] we found an experimental verification for a direct CCAAT box tandem repeat accompanied by an Sp1 binding site. The authors analyzed the highly conserved CCAAT box dimer in an inverted arrangement (distance of the CCAAT boxes Δ = 32) found in the promoter region of the human TOP2B gene. Lok et al. demonstrated that each single CCAAT box interacts with NF-Y and only the simultaneous disruption of the two CCAAT elements shuts down the promoter activity of this gene in a significant manner. In addition they demonstrated that the Sp1-binding GC box located upstream of the CCAAT tandem contributes to the TOP2B promoter activity in a synergistic manner. We have found this promoter region as part of our proximal intersection data set (co-localization of c-Fos and NF-YB). This example supports our hypothesis that the CCAAT box alone or in a tandem arrangement interacts with NF-Y. We think that these CCAAT modules define a very flexible enhancer/promoter interface. We summarize our hypothetical CCAAT dimer model in Fig 9. In Fig 9(a) we describe the basis regulatory module of the CCAAT dimer active in GM12878 cells. An additional gene regulatory influence could be performed if a distal/enhancer bound AP-1 can interact with a proximal CCAAT bound NF-Y promoter regions (Fig 9(b)). CCAAT boxes are found in the regulatory regions of 30% of genes [38]. Multiple CCAAT boxes are found in a large number of promoters and their genes are coding for so-called house-keeping genes, as well as inducible cell-cycle regulated genes [39]. We observe a specific subset of these genes which are regulated by the transcription factors c-Fos/AP-1 and NF-Y at the same time. A defined spacing of a direct CCAAT box repeat is found for this specific promoter class. In addition the biological process enrichment of chromatin remodeling processes support the specific function of the CCAAT dimer promoter class.

In human colorectal cancer cells it was shown that the transcription factor SOX9 binds to some cell cycle regulatory genes as a cofactor of NF-Y through the CCAAT motif [40]. This example demonstrates the capability of NF-Y to form regulatory active protein complexes. It was shown by Furumatsu et al. that SOX9 and p300 can interact [41]. We hypothesize that our CCAAT dimer model that is summarized in Fig 9 could be valid also for other transcription factors like SOX9 (see Fig 9). At least the manual inspection of the cell cycle regulatory genes CCNB1, CCNB2, CDK1, and TOP2A, which are targeted by SOX9 [40], contain one or more CCAAT box direct repeats. Three out of these four genes are included in our gene-proximal data set.

The intersection of FOS and NF-Y ChIP-seq regions were reported for the cell line K562 [11, 18]. Dolfini et al. confirmed this observation for HeLa S3 and GM12878 cells [19]. In addition the authors observed a “double CCAAT arrangment” in these peaks which are not found in ChIP-seq experiments done for the transcription factors FOSL1 and FOSL2. In our study we confirmed the lack of AP-1 motifs in co-localized ChIP-seq regions bound by the transcription factors FOS and NF-Y for four different cell lines analyzed in the Encode project. In addition we have shown a novel regulatory relationship between the transcription factors c-Fos/AP-1 and NF-Y. We observe a clear distinction between c-Fos-bound enhancers, which exhibit a canonical AP-1 motif, and c-Fos-bound promoter regions, which are characterized by NF-Y-bound CCAAT boxes, preferably as a direct dimer with a distinct distance distribution. Moreover, we observed mutually exclusive occurrence of AP-1 motifs and the CCAAT box in c-Fos bound regions, indicating that there is a polar interaction between AP-1 motif imprinted enhancers and CCAAT-containing promoters, which might be subject to interference upon presence of the respective other motif in the wrong environment.

As discussed in the paper from Mercer and Mattick [12] we believe that these particular promoter class can be found in ChIP-seq experiments because of a specific cross-linking between these interacting distal and proximal regions (enhanceosome structure). This type of enhancer-promoter correspondence is highly dependent on the biological context and, thus, differs between the four cell lines that we have investigated in our study. In our study we have shown that these direct CCAAT box repeats which are interacting with FOS and NF-Y at the same time are regulating a Gene Ontology defined biological process called nucleosome assembly. Recently it was shown that NF-Y controls the efficiency of DNA replication directly in a non-transcriptional way [42]. This observation supports our results: after replication the new synthesized DNA need to pack to nucleosmes. The gene products that are related to this process are summarized in this biological process. The promoters of this defined pathway are highly enriched for CCAAT box direct repeats and show significantly shorter distances to AP-1 motif containing enhancers.

## Conclusions

In this study we propose a novel regulatory function of the transcription factor NF-Y in gene-proximal regions co-localized with the transcription factor c-Fos. These NF-Y binding sites are found in a specific CCAAT dimer configuration around a distance value of 31 bp, accompanied by a series of progressively decreasing maxima at every 10-11 bp in addition. More generally, our study shows how versatile direct and indirect transcription factor occupancy in ChIP-seq regions may be. A regulatory region is bound by a number of TFs and only a subset of these factor can be found on the sequence level. This novel regulatory module introduce an additional level of complexity: the explicit missing of a transcription factor binding sites. We demonstrate that this level can be used to differentiate between direct or cis-regulatory influence in comparison to trans-regulatory (protein-protein interaction) effects.

## Materials and Methods

### ENCODE ChIP-seq and DNase-seq data sets

All data sets used in this study were taken from the ENCODE project [9] and performed on the GRCh37 (hg19) reference genome. ChIP-seq and DNase-seq genome-wide location data for the cell lines HUVEC, HeLa S3, K562, and GM12878 were used in this study. For all data sets we made use of the ENCODE project defined table files (narrowPeak), which provide called peaks of signal enrichment bases on pooled, normalized data [43]. For the HUVEC cell line we used the ChIP-seq data obtained with a c-Fos antibody (GEO: GSM935585) and DNase-seq data (GEO: GSM816646). No NF-YB related data set was available for this cell line. The ENCODE data sets analyzed for HeLa S3 cells were two ChIP-seq data sets for c-Fos (GEO: GSM935317) and NF-YB (GEO: GSM935408), and one DNase-seq data set (GEO: GSM816643). For K562 cells we examined ChIP-seq data for c-Fos, NF-YB, and DNase-seq data linked to the following GEO accession numbers: GSM935355, GSM935429, and GSM816655, resp. Finally we studied ChIP-seq data for c-Fos (GEO: GSM935409), NF-YB (GEO: GSM935507) and DNase-seq (GEO: GSM816665) obtained from GM12878 cells.

### Definition of proximal and distal regulatory regions

We classified the corresponding ENCODE ChIP- or DNase-seq data into distal or proximal regions depending on their genomic annotations: if an experimentally identified genomic interval was overlapping the minus 1000 bp region (-1kb) upstream of a RefSeq annotated transcription start site (TSS), including the first exon, we defined this interval as a proximal region. Each interval, which did not overlap the corresponding -1 kb upstream region or its related transcription unit was defined as a distal region. For the RefSeq data we used the UCSC genome browser provided download (05/14/2014). Both the ENCODE ChIP-/DNase-seq data and the RefSeq annotation used here were based on GRCh37 (hg19) reference genome annotation.

### Motif analysis using the TRANSFAC database

We used the TRANSFAC library (version 2013.3) together with the MATCH algorithm (Version 9.0) to analyze the different high-throughput data sets. We made use of all vertebrate matrices in this profile, which were altogether 2176 PWWs. For all matrices in this library we chose a matrix similarity score (MSS) threshold of 0.5. The core similarity score (CSS) threshold was set to 0 (for details see references [20, 22]).

### Performance analysis workflow

We developed a workflow to analyze experimentally determined regulatory sequence sets with PWMs stored in the TRANSFAC database. For each ChIP-seq data set under study, we removed all overlapping intervals from the DNase-seq data set of the same cell line and used the remaining genomic intervals as specific control set. We received four distinct cell type specific data sets: Two ChIP-seq data sets for c-Fos and NF-YB, resp., and two DNase-seq data sets as control for the c-Fos or the NF-YB set. We counted each ChIP-seq interval as a true positive (TP), when it contained at least one PWM match above the set threshold, otherwise it was classified as a false negative (FN). Likewise each DNase-seq region with at least one PWM match above the chosen threshold was counted as a false positive (FP), otherwise as a true negative (TN). For each of the PWMs we monitored TP, TN, FP and FN at gradually decreasing thresholds from the highest score (1.0) to 0.5. To determine the performance of a PWM, we made use of the classical receiver operator characteristic (ROC) curves by plotting the true positive rate (TPR: $TPR=\frac{TP}{TP+FN}$) against the false positive rate (FPR: $FPR=\frac{FP}{FP+TN}$). To summarize the classification efficiency represented by one ROC curve, we calculated the corresponding area under curve (AUC) values using R [44]. We computed Matthews Correlation Coefficient (MCC: $MCC=\frac{\left(TP\cdot TN)-(FP\cdot FN\right)}{\sqrt{\left(FP+TP)\cdot (TP+FN)\cdot (TN+FN)\cdot (TN+FP\right)}}$) to find the optimal MSS threshold for each PWM.

### De novo sequence motif discovery

*De novo* motif searching was performed with the MEME version 4.9.1 [45]. For the relevant ChIP-seq data, which are annotated as genomic location defined by their chromosomal locations, we retrieved the corresponding complete sequences in FASTA format. For each MEME run we chose the following parameterization: -dna, -revcomp, which instructs MEME to analyze DNA sequences and to consider both strands. To optimize the run time, we limited the MEME motif search to maximally 10 motifs (-nmotifs 10) with a minimal motif width of 5 but not more than 15 (-maxw 15). Finally, we chose the ‘zoops’ MEME parameter as the model for the distribution of motifs sites in our sequence set. Using this option MEME assumes that each sequence may contain at most one occurrence of each motif.

### Distance calculation of CCAAT box related tandem arrangements

CCAAT boxes were searched for as regular expressions. For all CCAAT boxes in a tandem arrangement, we computed the edge-to-edge distance. For concordant CCAAT boxes, the corresponding distance situation can be displayed as follows: 5’-CCAAT-3’…5’-CCAAT-3’ for the plus strand instance or 5’-ATTGG-3’…5’-ATTGG-3’ for its reverse complement. Other configurations (inverted/everted repeats) were not separately considered.

### Distance preferences between distal and proximal ChIP-seq regions

For the distance analysis between putative enhancer regions (distal ChIP-seq intervals) and their proximal or putative promoter regions, we also calculated an edge-to-edge distance. For each promoter region we determined all available upstream enhancer regions with the same chromosomal annotation. For all possible upstream regions we calculated the distance by subtracting the enhancer end position from the start position of the corresponding promoter region. Correspondingly, we subtracted all start positions of all downstream located enhancers from the same chromosome from the end position of the closest promoter element. From all the distances we determined the closest enhancer, either up- or downstream, for each promoter region. The distance is calculated from the first C of the first CCAAT to the first C of the second CCAAT box.

### GO analysis using GREAT

Proximal unified ChIP-seq intervals were submitted to the GREAT web service [27]. We chose the standard parameters of the workflow Basal plus extension based on the whole-genome background set. The gene regulatory domain definition used the following parameters: proximal extension 5 kb, downstream extension 1 kb, plusDistal up to 1000 kb.

### Repeat mapping

The repeating elements identified by RepeatMasker [46] are download from the UCSC (URL:http://www.hgdownload.cse.ucsc.edu/goldenPath/hg19/database/rmsk.txt.gz). We filter this file for all Long Terminal Repeat (LTRs) elements which resulted in 717,656 different LTR regions.

### p300 data

We identified p300 interfering regions using the ENCODE Transcription Factor Binding Track cluster(URL:http://hgdownload.cse.ucsc.edu/goldenPath/hg19/database/wgEncodeRegTfbsClusteredV3.txt.gz). Totally this file contains 138,836 p300 interfering regions.

### Co-localization analysis

For the co-localization analysis of ChIP-seq regions, we used the University of California Santa Cruz (UCSC) Table Browser tool collection [47].

## Supporting Information

S1 Fig ROC curves for characterizing c-Fos bound genomic intervals by AP-1 sequence motifs. c-Fos ChIP-seq data sets obtained from HUVEC (black curves), HeLa S3 (green), K562 (red) and GM12878 cells (blue) were analyzed with the TRANSFAC matrix V$JUNDM2_04 (M02876), using non-overlapping sets of DNase I hypersensitive sites from the same cell lines as control. Top inset: Logo plot representing the AP-1 motif detected by matrix V$JUNDM2_04 (top); bottom inset: Plot of Matthews correlation coefficient (MCC) against the applied threshold of the matrix similarity score (MSS) calculated by the Match program. The vertical line together with the yellow circle point to the MSS threshold used for detecting potential AP-1 sites in the further analyses. (A) Results for distal, (B) for proximal regions.
(TIF)

S2 Fig ROC curves for characterizing c-Fos-bound genomic intervals by NF-Y binding motifs. c-Fos ChIP-seq data sets obtained from HUVEC (black curves), HeLa S3 (green), K562 (red) and GM12878 cells (blue) were analyzed with the TRANSFAC matrix V$NFY_01 (M00287), using non-overlapping sets of DNase I hypersensitive sites from the same cell lines as control. Top inset: Logo plot representing the NF-Y binding motif detected by matrix V$NFY_01 (top); bottom inset: Plot of Matthews correlation coefficient (MCC) against the applied threshold of the matrix similarity score (MSS) calculated by the Match program. The vertical line together with the yellow circle point to the MSS threshold used for detecting potential AP-1 sites in the further analyses. (A) Results for distal, (B) for proximal regions.
(TIF)

S3 Fig Motif enrichment for the HUVEC cell line. Top five enriched motif for three different proximal (P1-P3) and distal (E1-E3) sample sets are shown. The used sample size was set to 657 (sample size of proximal c-Fos precipitated regions for the HeLa S3 cell line, see Table 4).
(TIF)

S4 Fig Motif enrichment for the HeLa S3 cell line. Top five enriched motif for proximal (P) and distal (E1-E3) sample sets are shown. The used sample size was set to 657 (sample size of proximal c-Fos precipitated regions for the HeLa S3 cell line, see Table 4).
(TIF)

S5 Fig Motif enrichment for the K562 cell line. Top five enriched motif for proximal (P1-P3) and distal (E1-E3) sample sets are shown. The used sample size was set to 657 (sample size of proximal c-Fos precipitated regions for the HeLa S3 cell line, see Table 4).
(TIF)

S6 Fig Motif enrichment for the GM12878 cell line. Top five enriched motif for proximal (P1-P3) and distal (E) sample sets are shown. The used sample size was set to 657 (sample size of proximal c-Fos precipitated regions for the HeLa S3 cell line, see Table 4).
(TIF)

S7 Fig Motif enrichment of c-Fos(only, proximal, AP-1+) sequence set. Motif discovery using MEME confirmed the predominant role of AP-1 consensus motifs in these c-Fos(only, proximal AP-1+) sequences.
(TIF)

S8 Fig Distance distribution of distal and proximal regions bound either by c-Fos or NF-YB. Proximal regions interacting with both c-Fos and NF-YB show significant shorter distance relations to c-Fos(only) bound distal regions (left) compared to NF-YB(only) bound distal regions (right).
(TIF)

S1 Table Distal and proximal gene regulatory regions derived by the Encode project. Shown are the number of genomic intervals bound by c-Fos or NF-YB in the indicated cell lines, retrieved from the corresponding data sets of the ENCODE repository. For the HUVEC cell lines no NF-YB ChIP-seq data is available (NA).
(TIF)

S2 Table Gene ontology enrichment of the Inter(c-Fos, NF-YB) group. The top 20 significantly enriched gene ontology biological process categories for proximal c-Fos and NF-YB co-localizing ChIP-seq regions are shown. 1724 proximal genomic intervals were analyzed (see Methods for details).
(TIF)

S3 Table Gene Ontology enrichment of the Inter(CCAAT2+) sequence set. The top 20 significantly enriched gene ontology biological process categories for c-Fos and NF-YB co-localizing ChIP-seq regions which contain at least one CCAAT dimer repeat were used as input set. 869 genomic intervals were analyzed (see Methods for details).
(TIF)

S4 Table Gene Ontology enrichment of the Inter(CCAAT2+,p300) sequence set. The top 20 significantly enriched gene ontology biological process categories for c-Fos and NF-YB co-localizing ChIP-seq regions which contain at least one CCAAT dimer repeat and are overlapping with ENCODE derived p300 ChIP-seq intervals were used as input set. 471 genomic intervals were analyzed (see Methods for details).
(TIF)

S1 File Gene-proximal c-Fos(only) data set. (BED)

S2 File Gen-proximal c-Fos and NF-YB co-localization data set. (BED)

S3 File Gen-proximal NF-YB(only) data set. (BED)

S4 File Gen-distal c-Fos(only) data set. (BED)

S5 File Gen-distal c-Fos and NF-YB co-localization data set. (BED)

S6 File Gen-distal NF-YB(only) data set. (BED)

S7 File Gene-proximal regions interfering with c-Fos and NF-YB, filtered CCAAT box direct repeats. (BED)

S8 File Gene-proximal regions interfering with c-Fos and NF-YB, filtered CCAAT box direct repeats and p300 co-localization. (BED)
