# Supplementary figures and images for: NF-Y Binding Site Architecture Defines a C-Fos Targeted Promoter Class
Source: PLoS One. 2016 Aug 12;11(8):e0160803. doi: 10.1371/journal.pone.0160803 (PMC4982600; doi:10.1371/journal.pone.0160803)

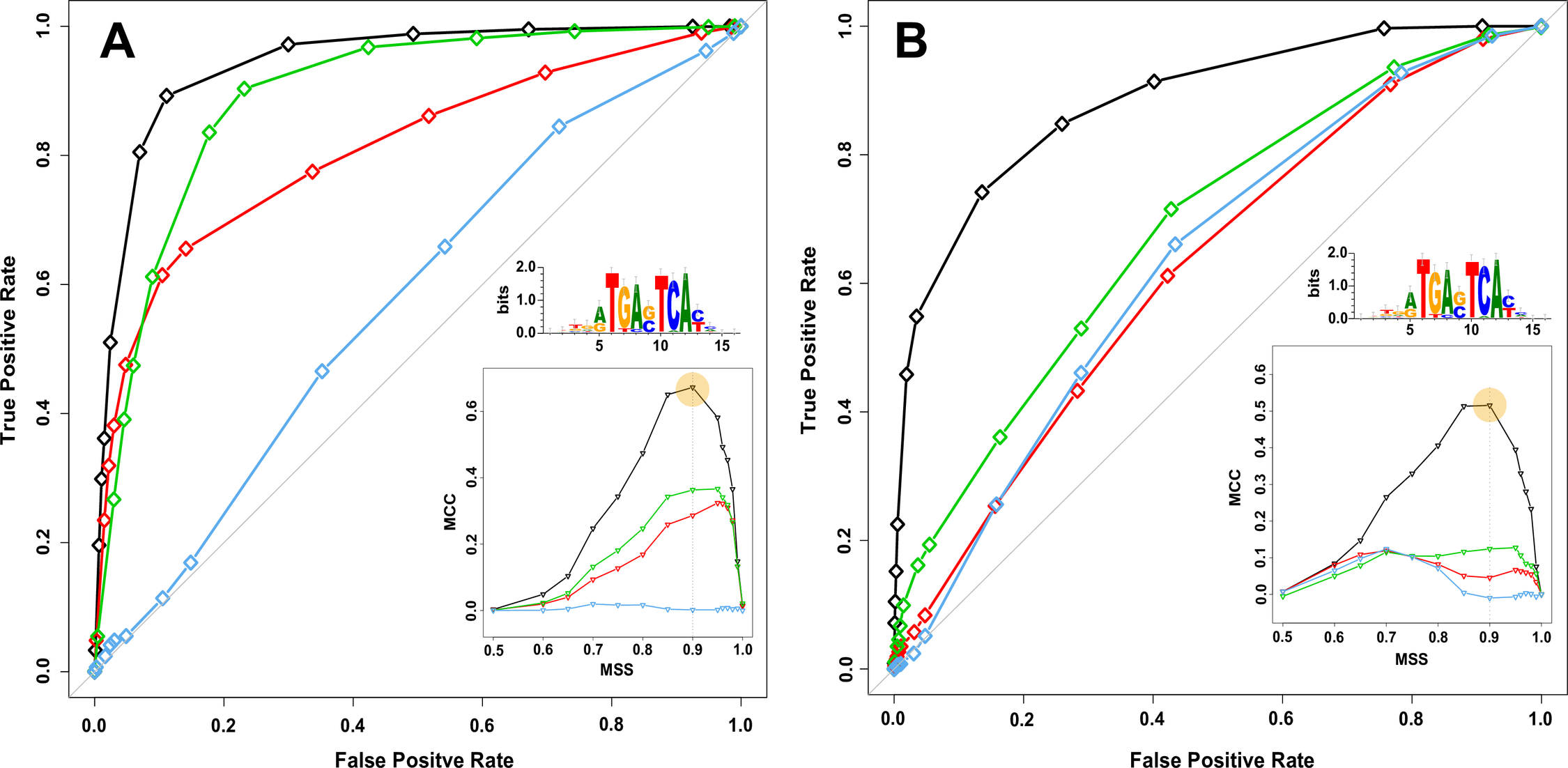

Supplement: S1 Fig — c-Fos ChIP-seq data sets obtained from HUVEC (black curves), HeLa S3 (green), K562 (red) and GM12878 cells (blue) were analyzed with the TRANSFAC matrix V$JUNDM2_04 (M02876), using non-overlapping sets of DNase I hypersensitive sites from the same cell lines as control. Top inset: Logo plot representing the AP-1 motif detected by matrix V$JUNDM2_04 (top); bottom inset: Plot of Matthews correlation coefficient (MCC) against the applied threshold of the matrix similarity score (MSS) calculated by the Match program. The vertical line together with the yellow circle point to the MSS threshold used for detecting potential AP-1 sites in the further analyses. (A) Results for distal, (B) for proximal regions. (TIF) [file pone.0160803.s001.tif]

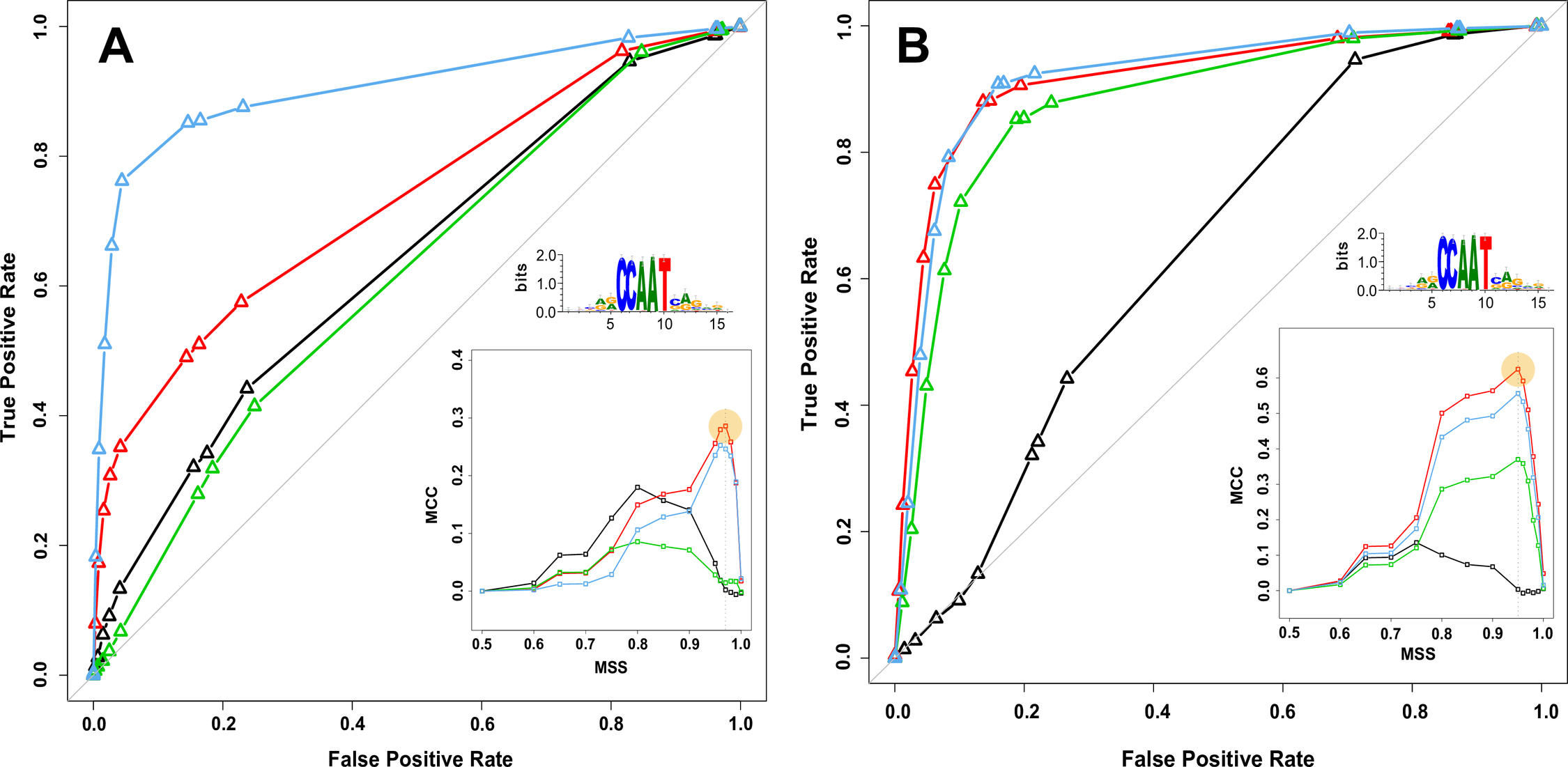

Supplement: S2 Fig — c-Fos ChIP-seq data sets obtained from HUVEC (black curves), HeLa S3 (green), K562 (red) and GM12878 cells (blue) were analyzed with the TRANSFAC matrix V$NFY_01 (M00287), using non-overlapping sets of DNase I hypersensitive sites from the same cell lines as control. Top inset: Logo plot representing the NF-Y binding motif detected by matrix V$NFY_01 (top); bottom inset: Plot of Matthews correlation coefficient (MCC) against the applied threshold of the matrix similarity score (MSS) calculated by the Match program. The vertical line together with the yellow circle point to the MSS threshold used for detecting potential AP-1 sites in the further analyses. (A) Results for distal, (B) for proximal regions. (TIF) [file pone.0160803.s002.tif]

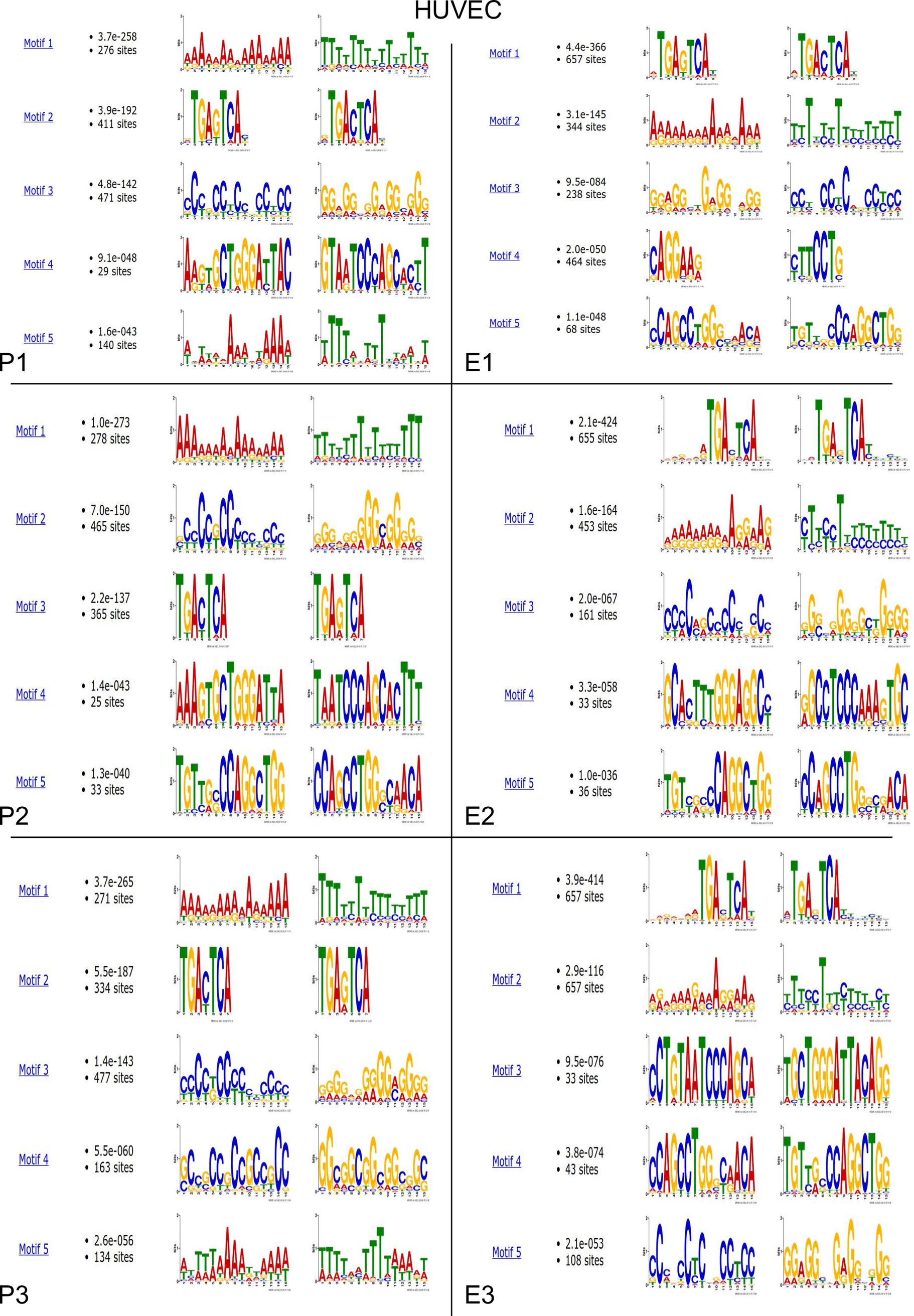

Supplement: S3 Fig — Top five enriched motif for three different proximal (P1-P3) and distal (E1-E3) sample sets are shown. The used sample size was set to 657 (sample size of proximal c-Fos precipitated regions for the HeLa S3 cell line, see Table 4). (TIF) [file pone.0160803.s003.tif]

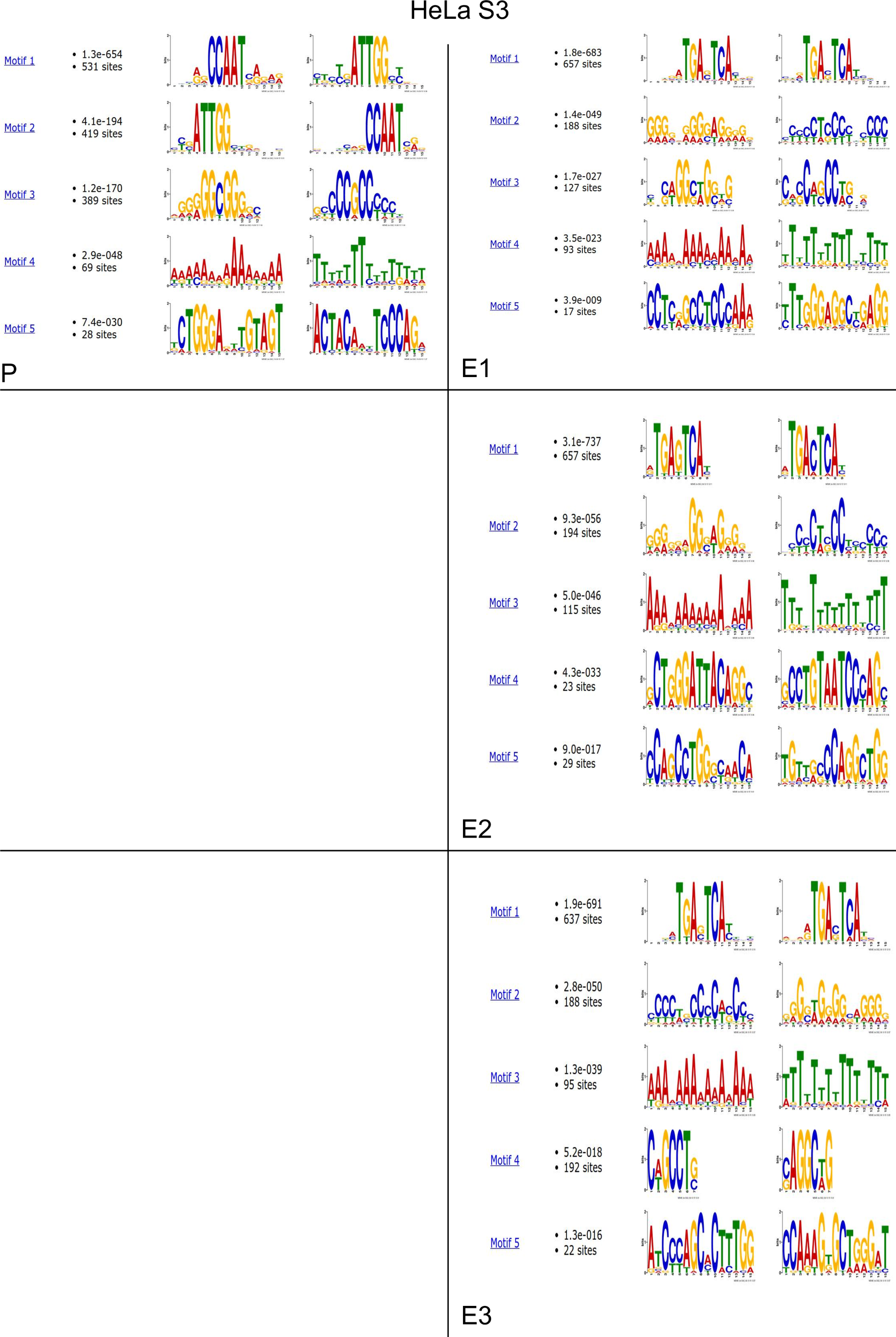

Supplement: S4 Fig — Top five enriched motif for proximal (P) and distal (E1-E3) sample sets are shown. The used sample size was set to 657 (sample size of proximal c-Fos precipitated regions for the HeLa S3 cell line, see Table 4). (TIF) [file pone.0160803.s004.tif]

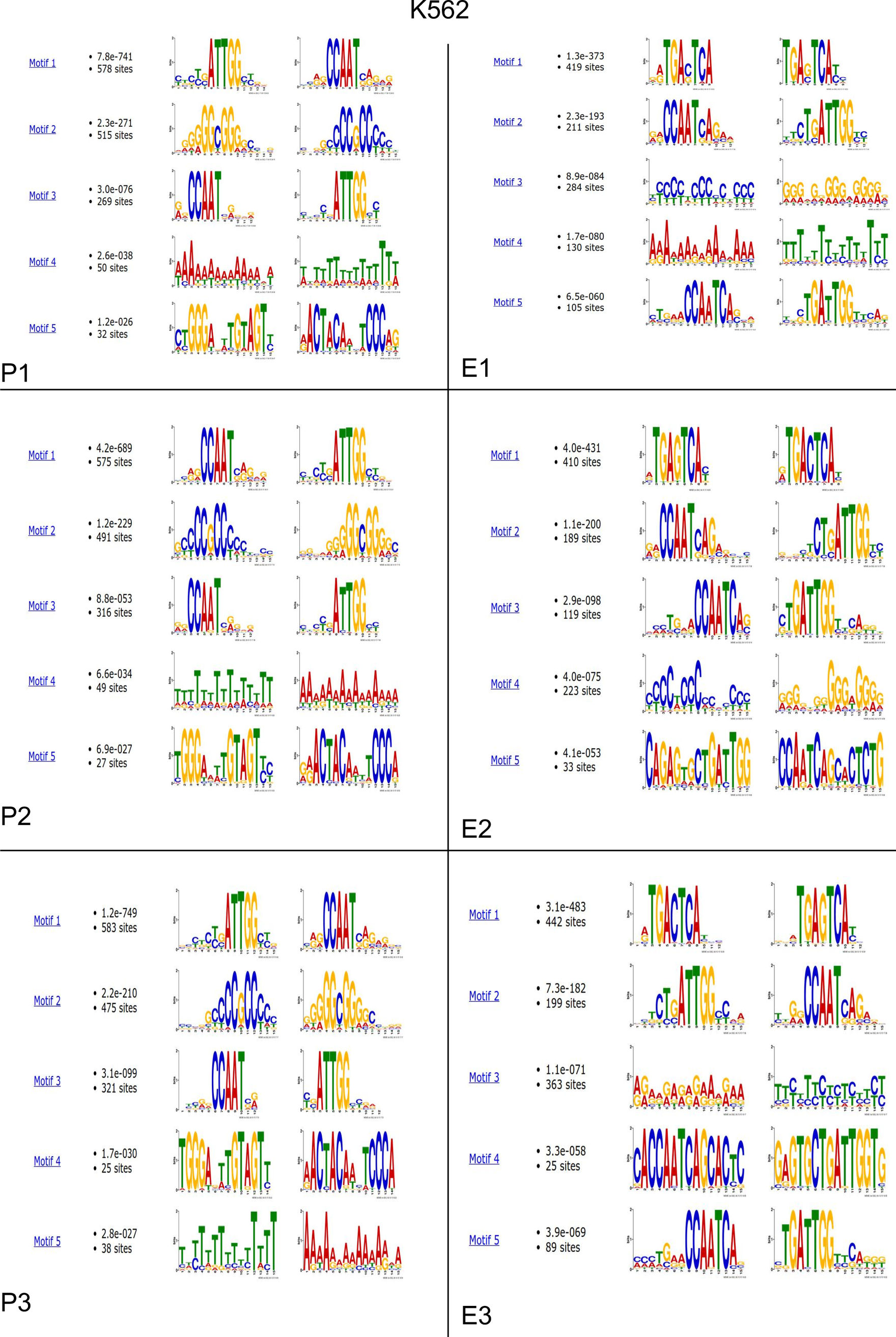

Supplement: S5 Fig — Top five enriched motif for proximal (P1-P3) and distal (E1-E3) sample sets are shown. The used sample size was set to 657 (sample size of proximal c-Fos precipitated regions for the HeLa S3 cell line, see Table 4). (TIF) [file pone.0160803.s005.tif]

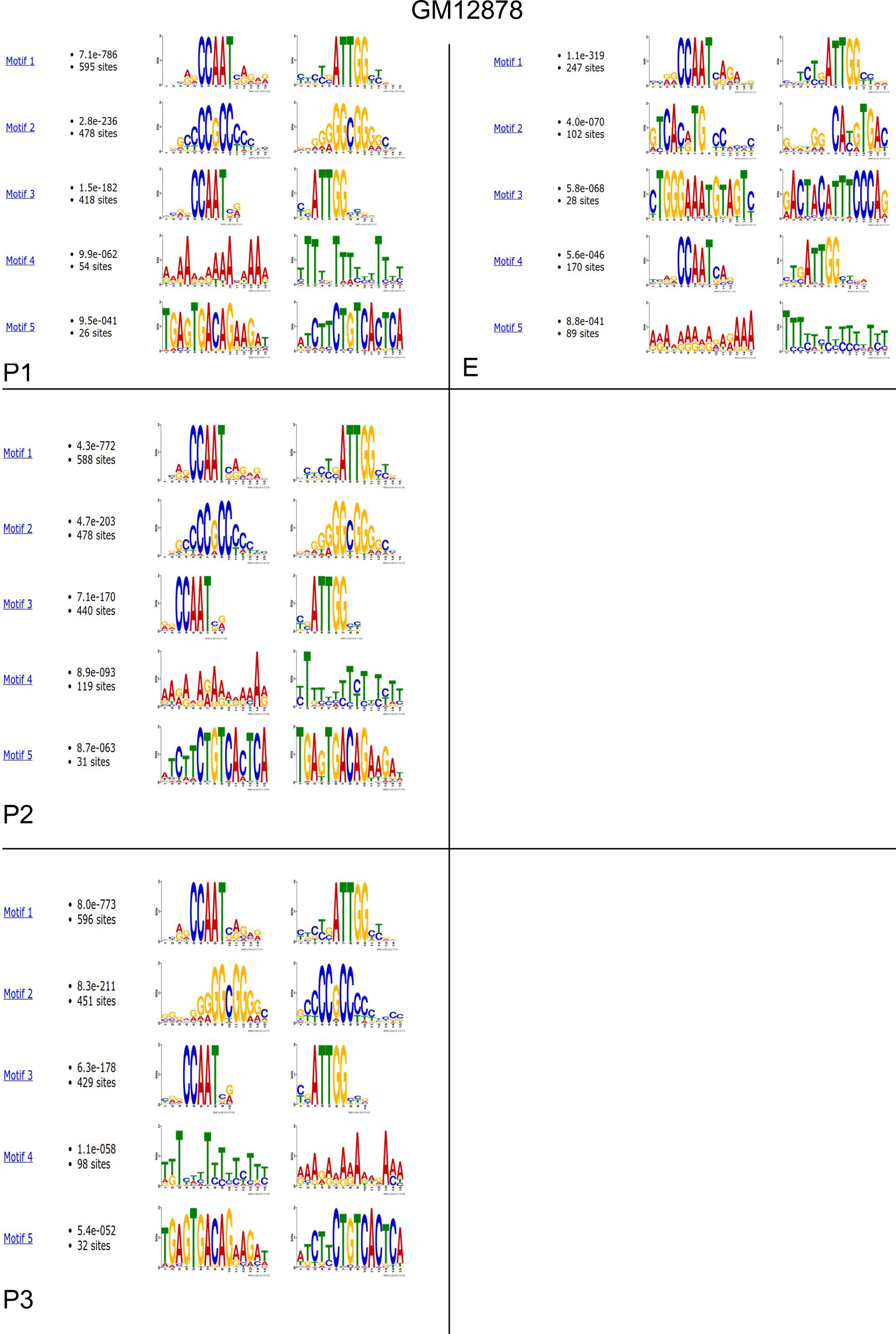

Supplement: S6 Fig — Top five enriched motif for proximal (P1-P3) and distal (E) sample sets are shown. The used sample size was set to 657 (sample size of proximal c-Fos precipitated regions for the HeLa S3 cell line, see Table 4). (TIF) [file pone.0160803.s006.tif]

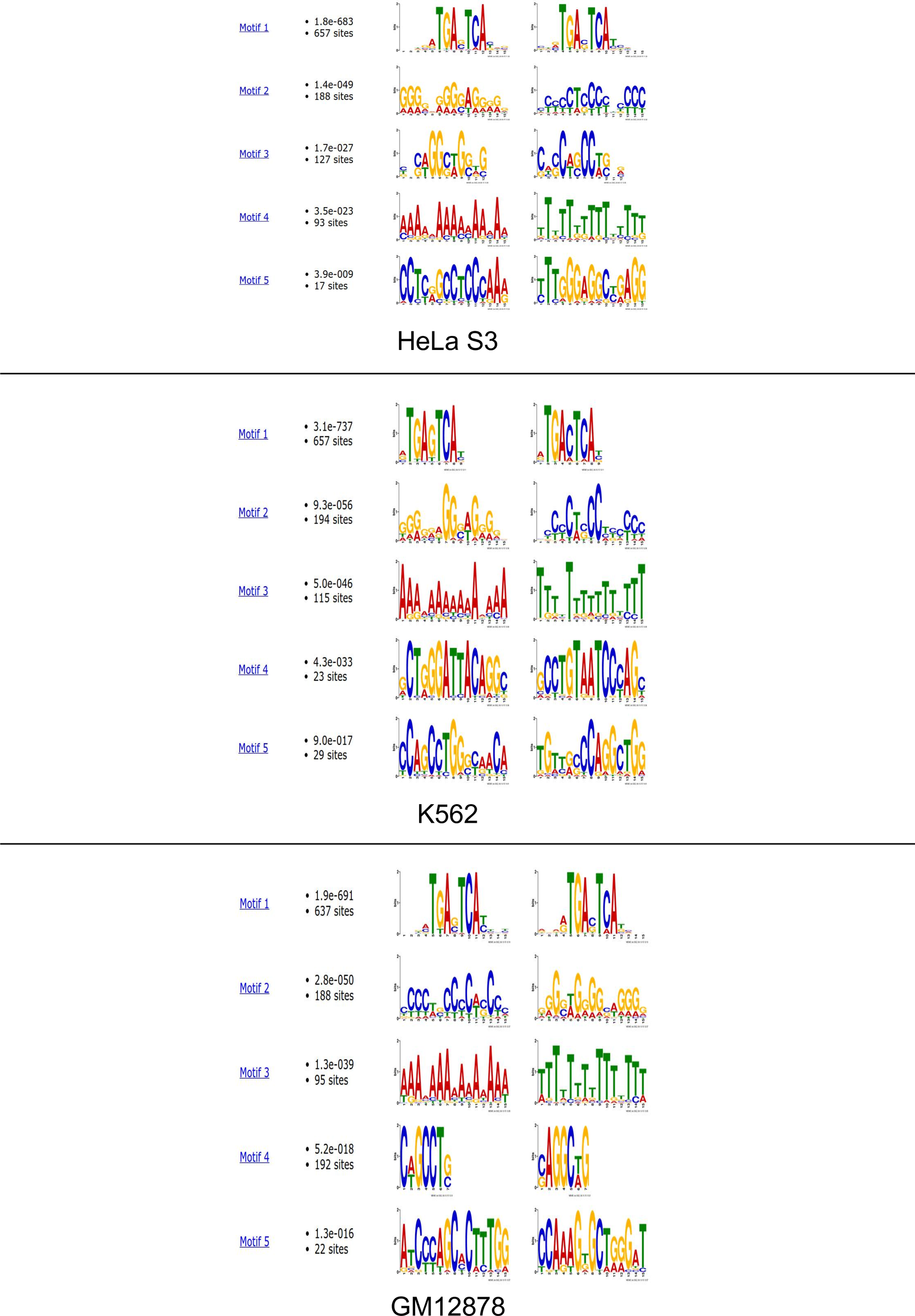

Supplement: S7 Fig — Motif discovery using MEME confirmed the predominant role of AP-1 consensus motifs in these c-Fos(only, proximal AP-1+) sequences. (TIF) [file pone.0160803.s007.tif]

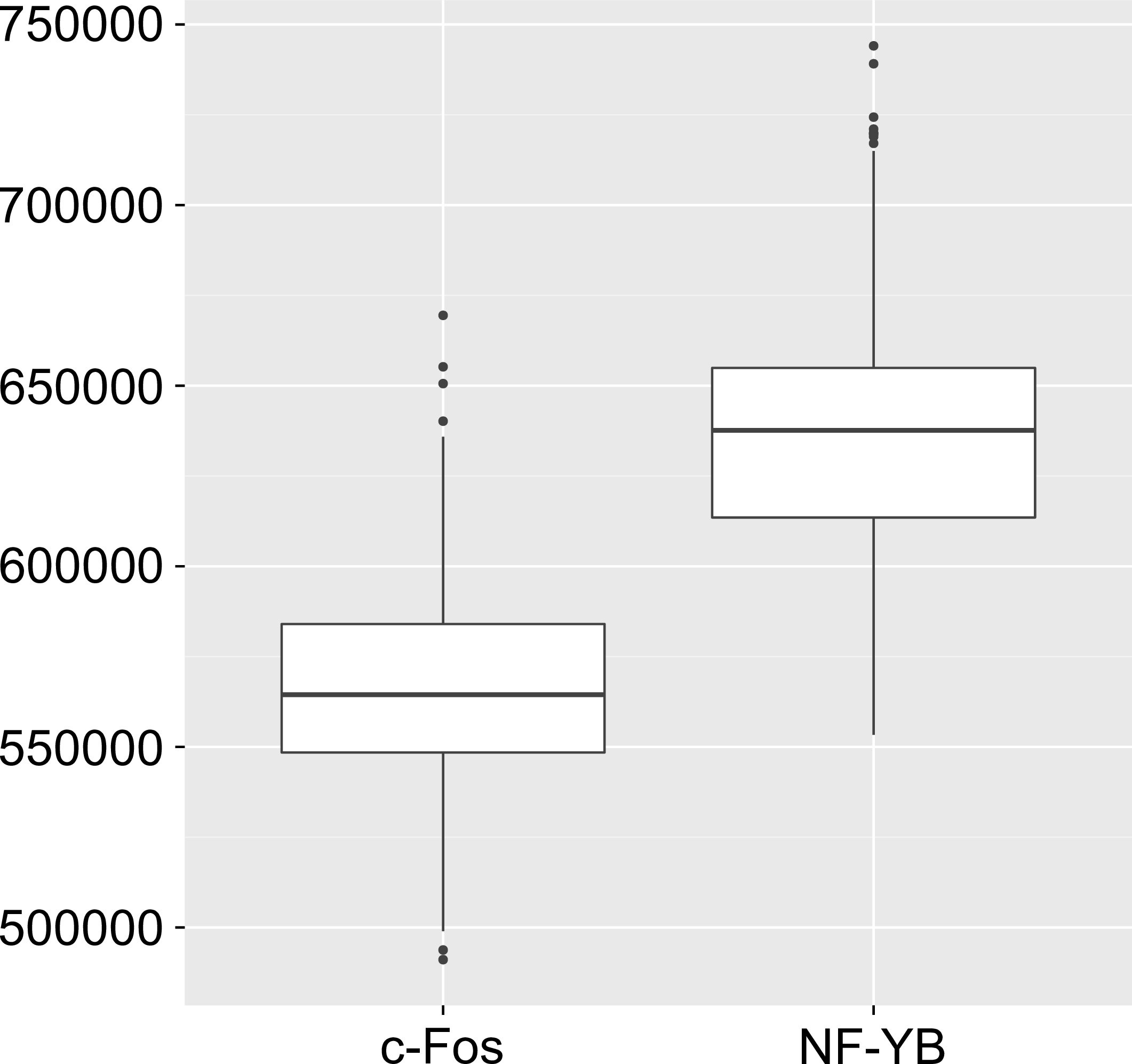

Supplement: S8 Fig — Proximal regions interacting with both c-Fos and NF-YB show significant shorter distance relations to c-Fos(only) bound distal regions (left) compared to NF-YB(only) bound distal regions (right). (TIF) [file pone.0160803.s008.tif]

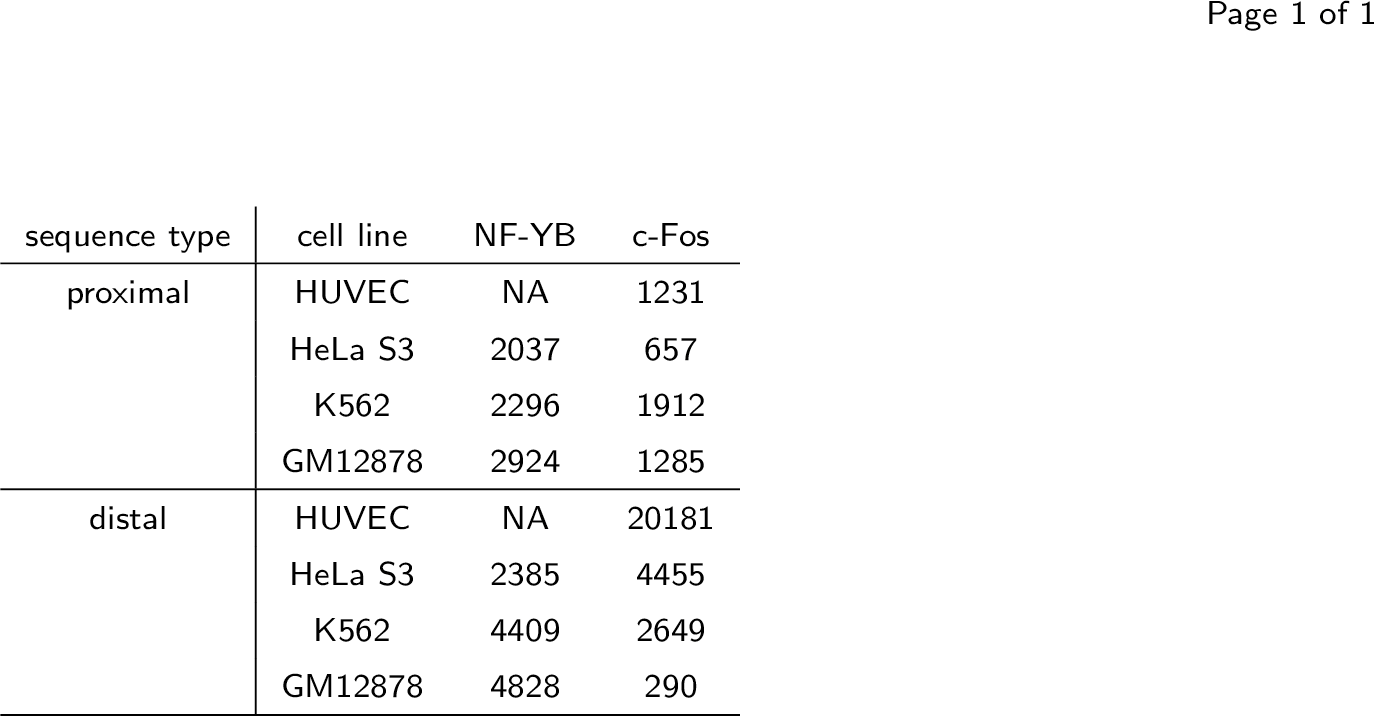

Supplement: S1 Table — Shown are the number of genomic intervals bound by c-Fos or NF-YB in the indicated cell lines, retrieved from the corresponding data sets of the ENCODE repository. For the HUVEC cell lines no NF-YB ChIP-seq data is available (NA). (TIF) [file pone.0160803.s009.tif]

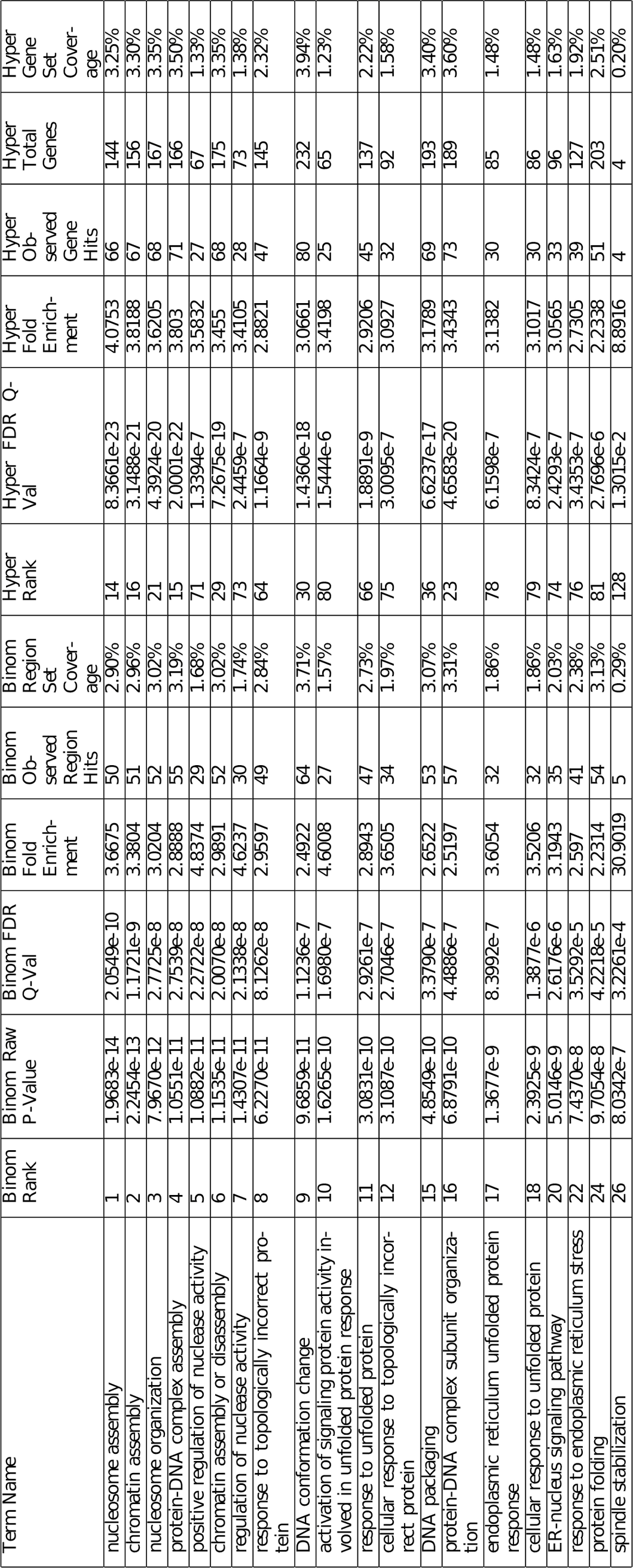

Supplement: S2 Table — The top 20 significantly enriched gene ontology biological process categories for proximal c-Fos and NF-YB co-localizing ChIP-seq regions are shown. 1724 proximal genomic intervals were analyzed (see Methods for details). (TIF) [file pone.0160803.s010.tif]

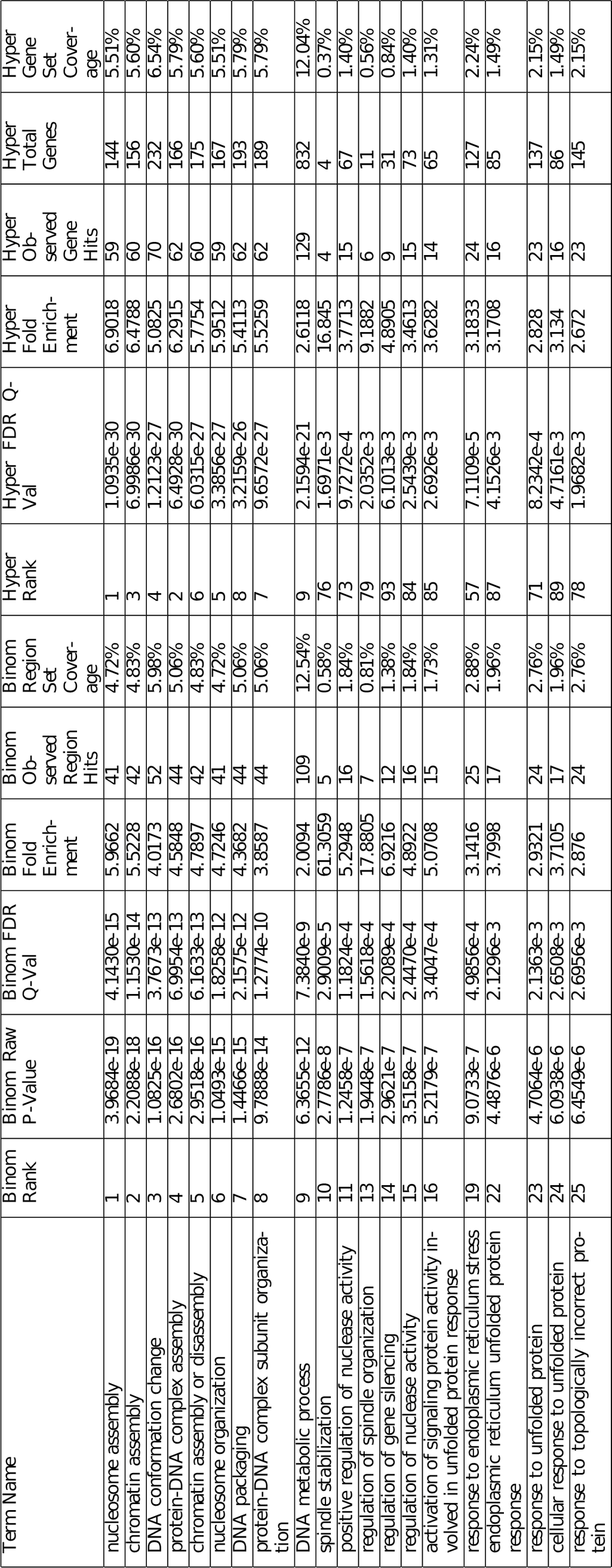

Supplement: S3 Table — The top 20 significantly enriched gene ontology biological process categories for c-Fos and NF-YB co-localizing ChIP-seq regions which contain at least one CCAAT dimer repeat were used as input set. 869 genomic intervals were analyzed (see Methods for details). (TIF) [file pone.0160803.s011.tif]

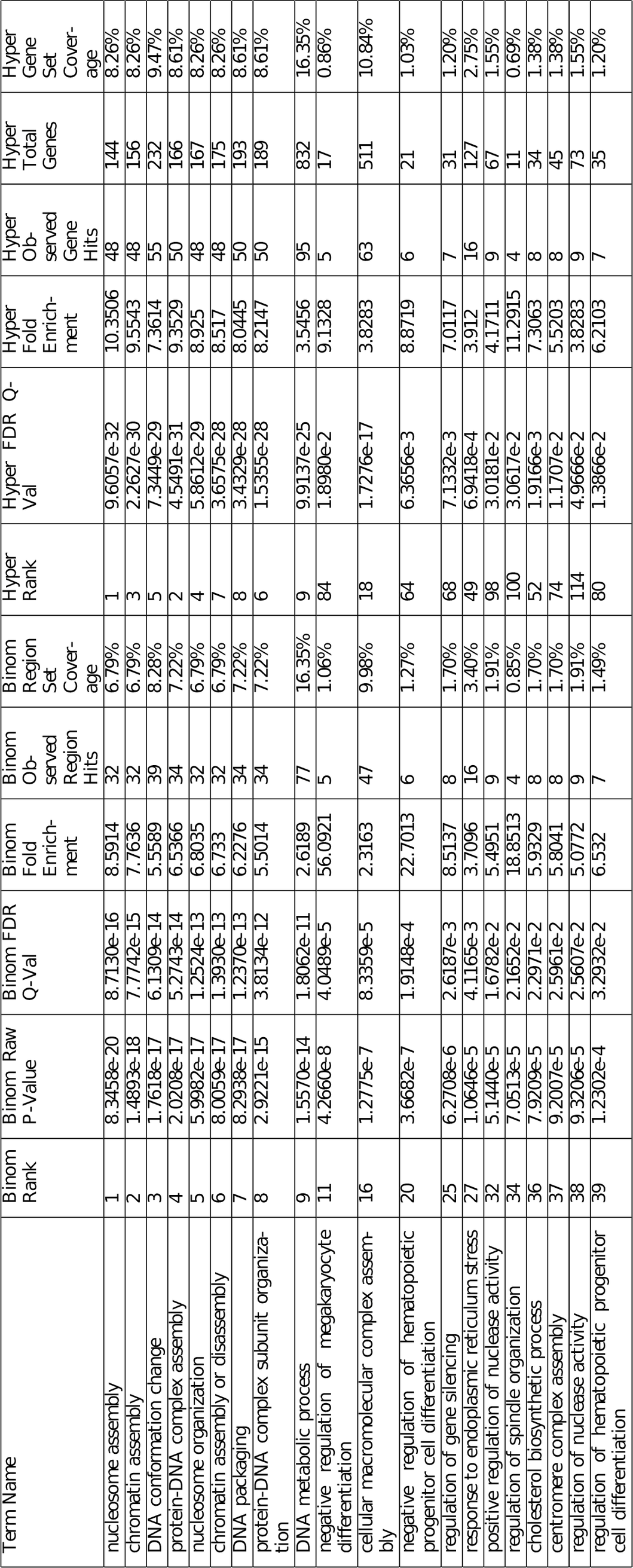

Supplement: S4 Table — The top 20 significantly enriched gene ontology biological process categories for c-Fos and NF-YB co-localizing ChIP-seq regions which contain at least one CCAAT dimer repeat and are overlapping with ENCODE derived p300 ChIP-seq intervals were used as input set. 471 genomic intervals were analyzed (see Methods for details). (TIF) [file pone.0160803.s012.tif]
